# [1,2,5]Thiadiazolo[3,4-*d*]Pyridazine as an Internal Acceptor in the D-A-π-A Organic Sensitizers for Dye-Sensitized Solar Cells

**DOI:** 10.3390/molecules24081588

**Published:** 2019-04-22

**Authors:** Timofey N. Chmovzh, Ekaterina A. Knyazeva, Ellie Tanaka, Vadim V. Popov, Ludmila V. Mikhalchenko, Neil Robertson, Oleg A. Rakitin

**Affiliations:** 1 N. D. Zelinsky Institute of Organic Chemistry, Russian Academy of Sciences, 119991 Moscow, Russia; tim1661@yandex.ru (T.N.C.); katerina_knyazev@ioc.ac.ru (E.A.K.); mlv@ioc.ac.ru (L.V.M.); 2 Nanotechnology Education and Research Center, South Ural State University, 454080 Chelyabinsk, Russia; popov.ioc@gmail.com; 3 EaStCHEM School of Chemistry, University of Edinburgh, Edinburgh EH9 3FJ, UK; Ellie.Tanaka@ed.ac.uk

**Keywords:** sulfur-nitrogen heterocycles, [1,2,5]thiadiazolo[3,4-*d*]pyridazine, dye-sensitized solar cells, power conversion efficiency

## Abstract

Four new D-A-π-A metal-free organic sensitizers for dye-sensitized solar cells (DSSCs), with [1,2,5]thiadiazolo[3,4-*d*]pyridazine as internal acceptor, thiophene unit as π-spacer and cyanoacrylate as anchoring electron acceptor, have been synthesized. The donor moiety was introduced into [1,2,5]thiadiazolo[3,4-*d*]pyridazine by nucleophilic aromatic substitution and Suzuki cross-coupling reactions, allowing design of D-A-π-A sensitizers with the donor attached to the internal heterocyclic acceptor not only by the carbon atom, as it is in a majority of DSSCs, but by the nitrogen atom also. Although low values of power conversion efficiency (PCE) were found, a few important consequences were identified: (i) poor PCE data can be attributed to high electron deficiency of the internal [1,2,5]thiadiazolo[3,4-*d*]pyridazine acceptor due to lower light harvesting by the dye; (ii) the manner in which the donor was attached to the internal acceptor (by carbon or nitrogen) did not play an essential role in the photovoltaic properties of the dyes; (iii) dyes based on the novel donor 2,3,4,4a,9,9a-hexahydro-1*H*-1,4-methanocarbazolyl and 9-(*p*-tolyl)-2,3,4,4a,9,9a-hexahydro-1*H*- carbazole moieties showed similar photovoltaic properties to dyes based on the well-known 4-(*p*-tolyl)-1,2,3,3a,4,8b-hexahydrocyclopenta[b]indolyl building block, which opens the door for further optimization potential of new dye families.

## 1. Introduction

In the recent years, solution processable solar cells, including dye-sensitized solar cells (DSSCs) [1,2,3,4], bulk heterojunction donor-acceptor blends [5,6], quantum dot solar cells [7,8], organic–inorganic hybrid perovskite solar cells [9,10,11], and tandem solar cells [12,13] have attracted much attention to in the search to produce low-cost electricity and portable energy. Among them, DSSCs, as a new kind of green energy device, display important properties such as easy fabrication, relatively low production cost, low toxicity, and good flexibility of molecular design [14,15,16]. Among all the components of DSSCs, the photosensitizers play a key role, being responsible for light harvesting and then electron transfer to a wide band gap semiconducting oxide (typically TiO_2_). Hence, the optoelectronic properties of sensitizing dyes are crucial to the photovoltaic performance of DSSCs. Therefore, the molecular engineering of sensitizing dyes is one of the most efficient routes by which to advance the performance of DSSCs. One of the most successful design strategies of metal-free organic dyes involves the use of D-π-A architectures, due to their easy synthesis and reliable performance [17,18,19,20]. Recently, Zhu and Tian proposed a new concept with a D-A-π-A configuration for designing a generation of stable and efficient organic sensitizers [21,22]. Compared to D-π-A dyes, D-A-π-A sensitizers, where the auxiliary acceptor is inserted between the donor and the π-bridge, showed broadened absorption, optimized energy levels, enhanced stability, and efficient intramolecular charge transfer for high performance DSSCs [23]. Some electron-withdrawing groups, such as diketopyrrolopyrrole [24], benzothiadiazole [25], selenadiazolopyridine [26], benzotriazole [27], quinoxaline [28], isoindigo [29], and many others, have been inserted into the D-A-π-A framework. However, so far, a deep understanding of the relationship between the structure of the internal acceptor group and the photovoltaic properties of the DSSCs is still lacking. For example, it has been found that D-A-π-A type dye molecules containing the more electron-withdrawing benzothiadiazole auxiliary unit show higher light-harvesting efficiency than those with benzotriazole moiety, thus resulting in an enhanced photocurrent (Jsc) of the corresponding DSSCs [30].

**Figure 1:**
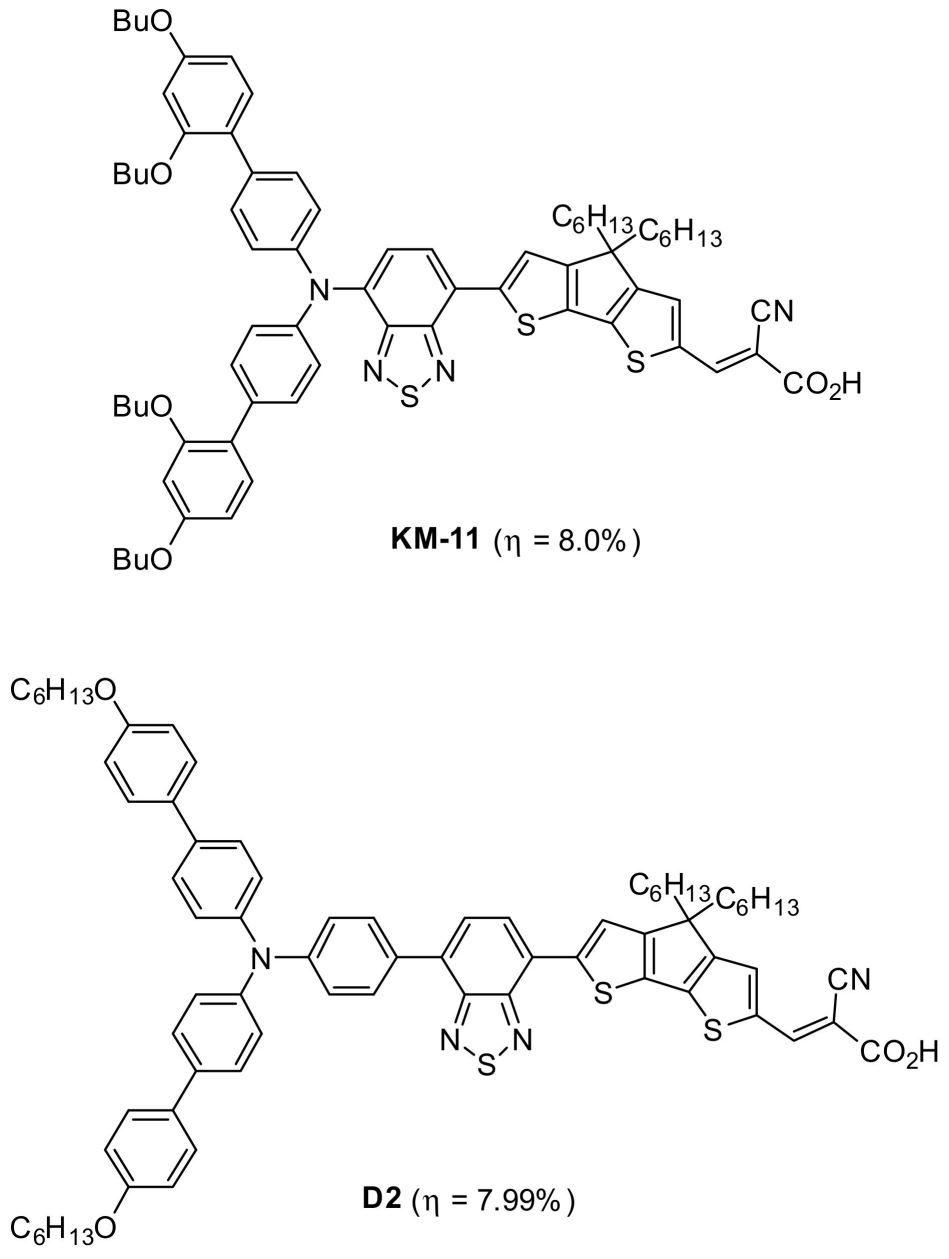
Comparing N- and C-donor attached D-A-π-A dyes.

There are a few examples of D-A-π-A dyes where the donor moiety is attached to the acceptor by the nitrogen atom. Comparison of the photovoltaic properties for two sensitizers of D-A-π-A structure, **KM-11** [31] and **D2** [32], (Figure 1) showed that there is no difference between them in photovoltaic efficiency, and the challenge to use a donor fragment attached by the nitrogen atom remains interesting. The introduction of the N-donor fragment into benzene fused with thiadiazole or similar heterocycles does not look simple and promising, since it requires use of palladium-catalyzed Buchwald-Hartwig or the rarely employed Ullman strategy (compared with introduction of a C-donor by similar palladium-catalyzed Suzuki or Stille cross-couplings). Nevertheless, this strategy looks quite promising for highly reactive S_N_Ar substitution reactions of electron-deficient heterocycles.

Recently, we reported the synthesis of the highly electron-deficient [1,2,5]thiadiazolo[3,4-*d*]pyridazine building block, which was evaluated as one of the strongest electron-acceptor systems [33]. It was found that substitution chemistry of 4,7-dibromo[1,2,5]thiadiazolo[3,4-*d*]pyridazine (aromatic nucleophilic and palladium-catalysed cross-couplings) was a powerful tool for the selective formation of various mono- and bis-derivatives of strong electron-accepting heterocycles. Conditions for selective aromatic substitution of one bromine atom by oxygen and nitrogen nucleophiles [34], as well as by Suzuki-Miyaura coupling by arylboronic acids, were found. These results have driven us to the conclusion that this might be a good basis for the synthesis of dyes for DSSCs.

**Figure 2:**
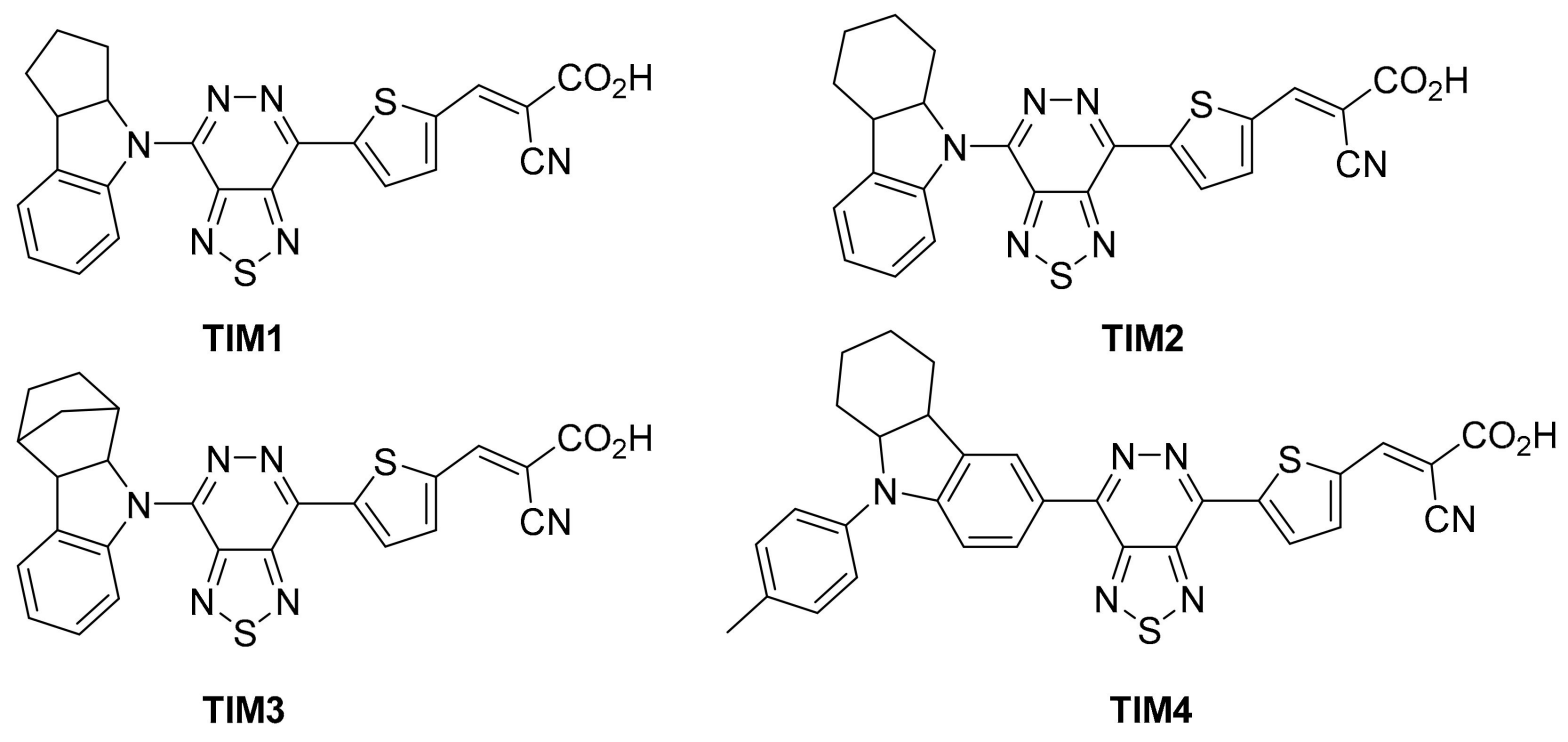
Chemical structures of the dyes synthesized.

In this work, we therefore aimed to obtain new organic dyes based on the [1,2,5]thiadiazolo[3,4-*d*]pyridazine unit. Taking into account the ease of introduction of N-nucleophiles into [1,2,5]thiadiazolo[3,4-*d*]pyridazine by nucleophilic aromatic substitution, we aimed to design and prepare D-A-π-A sensitizers with the donor attached to the internal heterocyclic acceptor not only by the carbon atom, as it is in a majority of DSSCs, but by the nitrogen atom also. It is known that an indoline group (in **TIM1**) has been proven to endow stronger electron-donating ability than other donor moieties, such as triphenylamine or carbazole [23]. We believed that introduction of similar cycloalkyl groups (such as hexahydrocarbazole in **TIM2** and **TIM4**, and tetrahydromethanocarbazole in **TIM3**) would also increase the donor ability in the corresponding dyes. Herein, the first few dyes using this new internal acceptor (Figure 2) were synthesized, characterized, and tested. The photo-physical and photo-electric properties of these dyes were systemically studied to identify useful design criteria for the discovery of further new dyes within this family.

## 2. Results and Discussion

### 2.1. Synthesis and Characterization

**Scheme 1:**
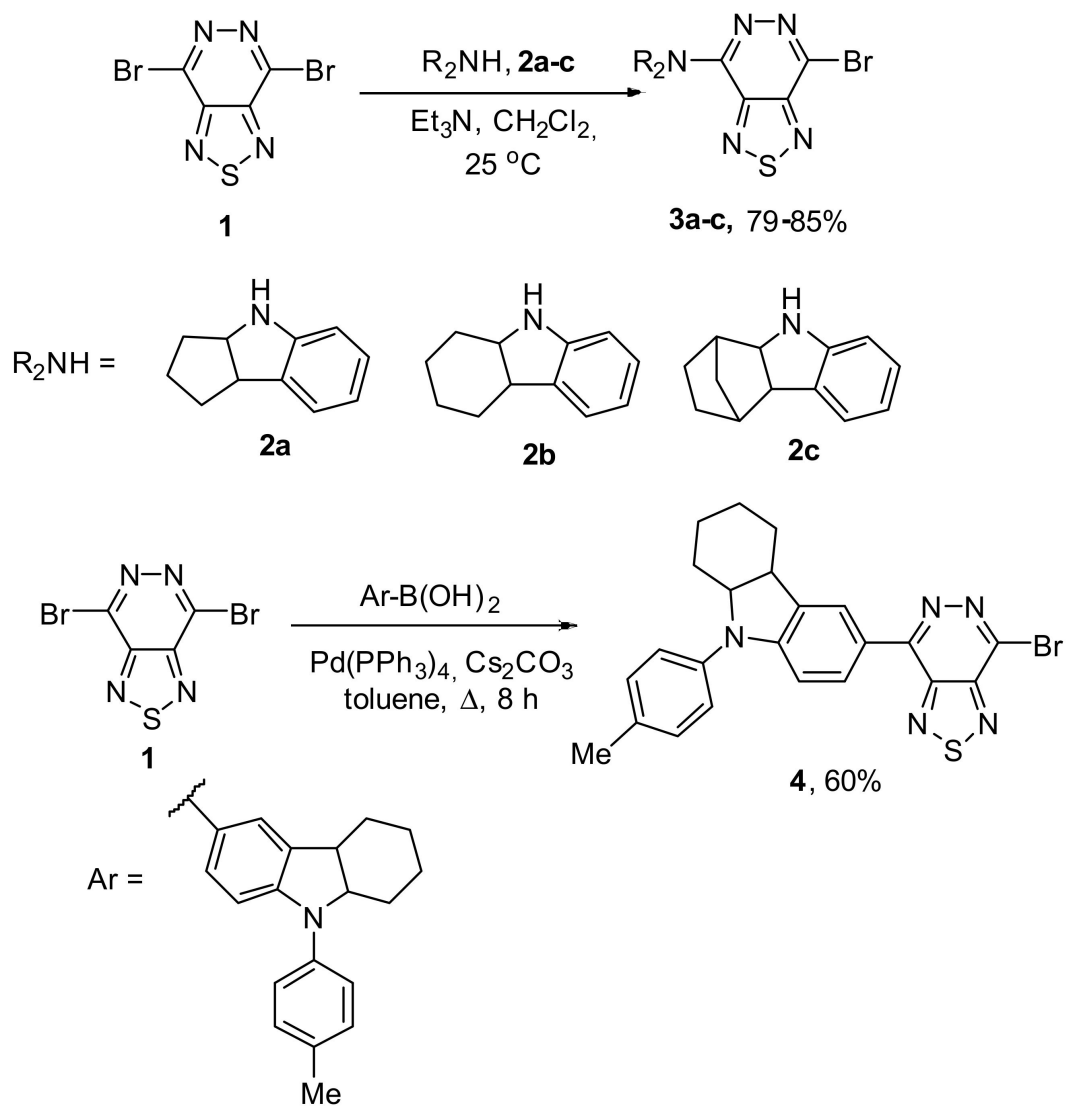
Synthesis of mono-adducts **3a**–**c** and **4**.

Mono-adducts **3a**–**c** and **4** were synthesized by nucleophilic aromatic substitution with appropriate amines [34] and by Suzuki cross-coupling reaction with boronic acid, following the procedures described [33] (Scheme 1).

**Scheme 2:**
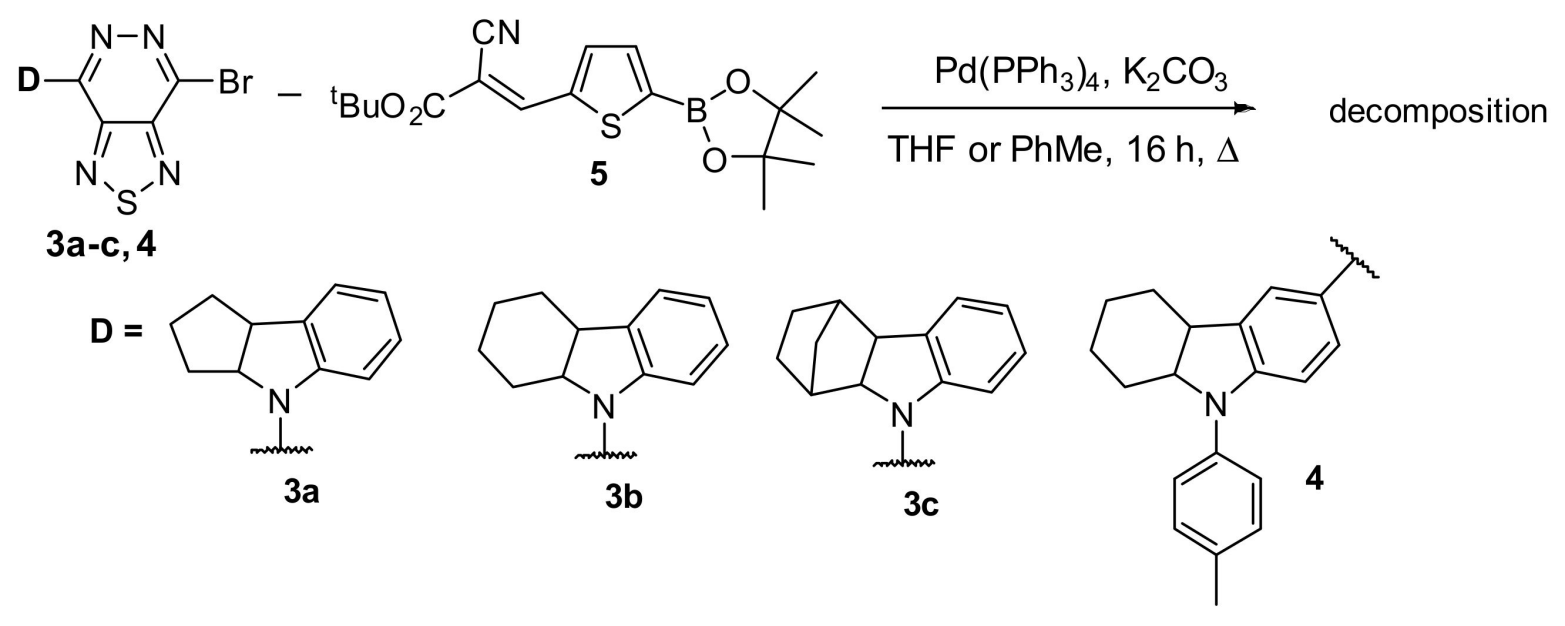
Reaction of mono-adducts **3a–c** and **4** with *tert*-butyl ester **5**.

Unexpectedly, the second Suzuki cross-coupling between mono-adducts **3a**–**c** and **4** with *tert*-butyl ester **5** in the presence of Pd(PPh_3_)_4_ as a catalyst and aqua solution K_2_CO_3_ in tetrahydrofurane (THF) or toluene, which was successfully used for 4-bromo[1,2,5]thiadiazolo[3,4-*c*]pyridines and 4-bromo[1,2,5]selenadiazolo[3,4-*c*]pyridines [26], failed, and only decomposition products were detected (Scheme 2).

**Scheme 3:**
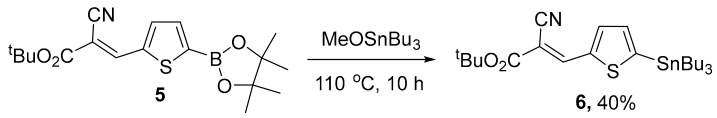
Synthesis of *tert*-butyl 2-cyano-3-(5-(tributylstannyl)thiophen-2-yl)acrylate **6**.

We assumed that mono-adducts are insufficiently stable by the action of water or a base (K_2_CO_3_) and the Stille coupling in non-aqueous and non-basic conditions may be successful. It was recently described that aryltributylstannanes can be easily prepared from arylboronic acids and tributyltin methoxide [35] We have found that thienylboronic ester **5** reacted with tributyltin methoxide under argon to afford thiophenetributylstannane **6** in moderate yield (Scheme 3).

**Scheme 4:**
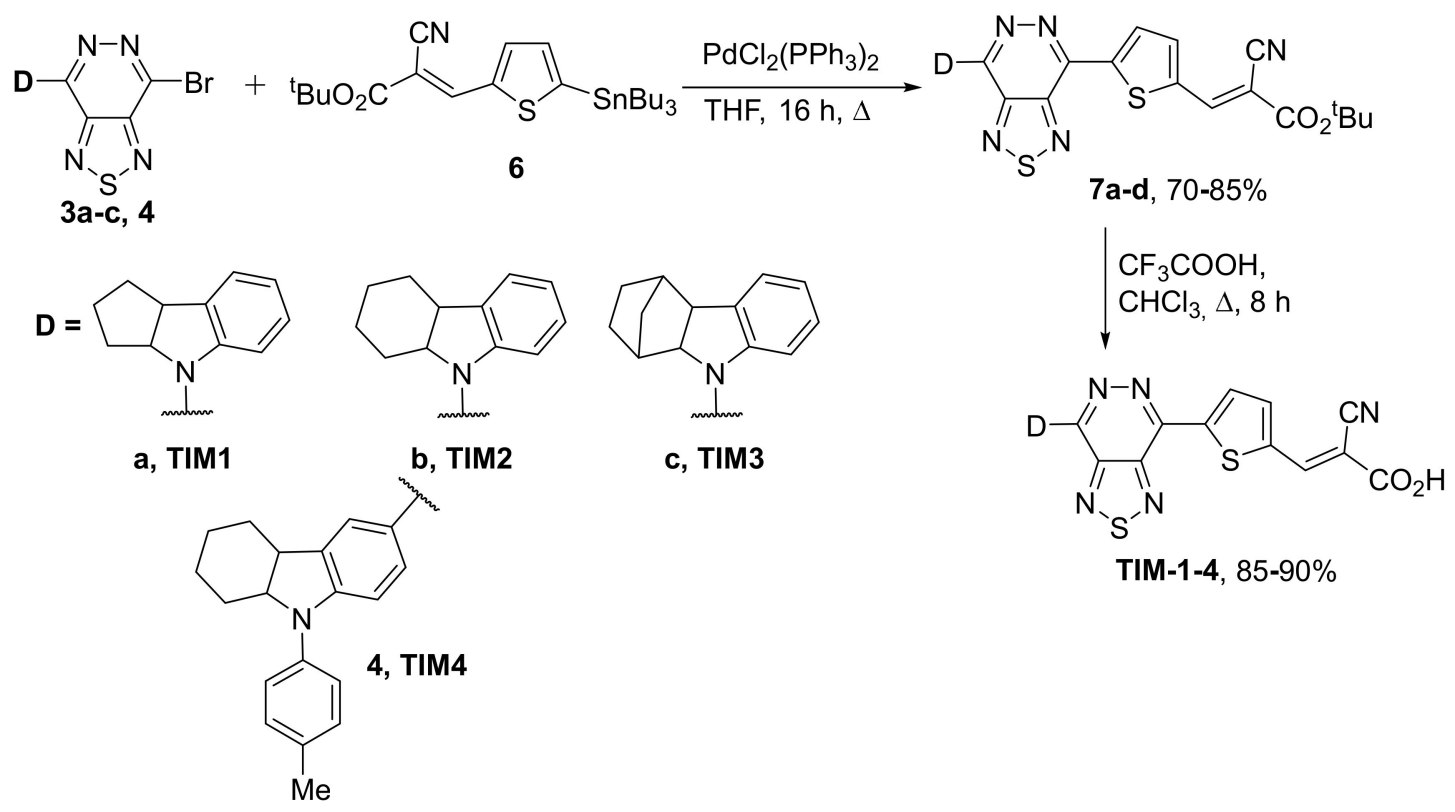
Synthesis of dyes.

Mono-adducts **3a**–**c** and **4** were subjected to Stille cross-coupling reactions with tributylstannane **6** in the presence of PdCl_2_(PPh_3_)_2_ as a catalyst in non-aqueous and non-basic conditions in THF, to afford bis-adducts **7a**–**d** in high yields. Final hydrolysis of compounds **7a**–**d** with CF_3_CO_2_H resulted in the formation of the target dyes in high yields (Scheme 4). All dyes were purified by column chromatography before measurements of the physical and electrochemical properties and solar cell device fabrication (characterization data including ^1^H- and ^13^C-NMR spectra for the compounds **6**, **7a**–**d**, TIM1–3 are shown in Supplementary Materials).

### 2.2. Photophysical and Electrochemical Properties

**Figure 3:**
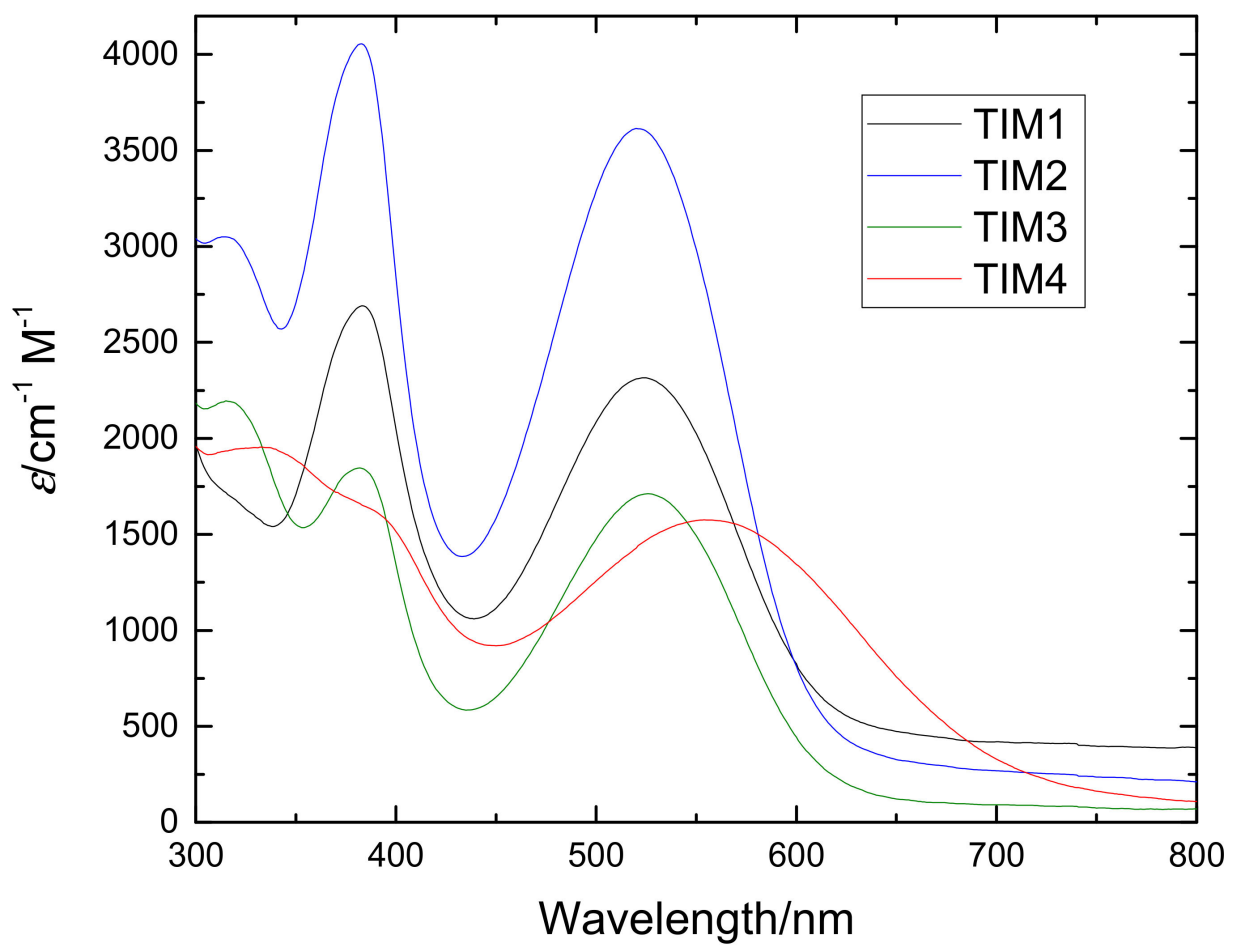
UV–visible absorption of the **TIM** series in EtOH at 5 × 10^−^^5^ mol mL^−^^1^.

The response region in sunlight for DSSCs is determined primarily by the UV–vis absorption of the sensitizer. Therefore, we initially characterized the spectral response of the **TIM** series in EtOH at 5 × 10^−5^ mol L^−1^ (Figure 3). The absorption peaks (λ_max_) and their corresponding molar absorption coefficients (ε) are listed in Table 1.

As shown in Figure 3, three compounds of the **TIM** series had two pronounced absorption maxima. Firstly, the absorption in the 380 nm area mainly corresponded to the π–π* electronic transition. For the **TIM4** compound, this maximum was weakly expressed, but the presence of a plateau in the region of 370–410 nm suggests the presence of the same electronic effects as for the other three dyes of the **TIM** series. Secondly, the absorption bands between 520–560 nm were assigned to an intramolecular charge transfer (ICT) process between the donor and anchor/acceptor group, which produced an efficient charge-separated excited state. We found that, for dyes in which the donor fragment is connected to the pyridazine ring by a nitrogen atom, both absorption maxima wavelengths differed only slightly. The size of the substituent in dihydroindole in the dye **TIM1–3** affected the absorption intensity: the highest extinction coefficient was observed for the derivative with a cyclohexane ring (**TIM2**), and the smallest for the skeleton derivative **TIM3**. The dye **TIM4**, in which the donor fragment was connected to the pyridazine cycle by a carbon atom, had a hypsochromic shift of the long-wavelength absorption maximum compared to the analogues with C–N bonds (**TIM1–3**). This indicates an increase in the conjugation chain in the **TIM4** compound, due to the presence of an additional *p*-tolyl group. It is worth noting that the extinction coefficient for this dye was also low, as for the derivative norbornene (**TIM3**). In general, the extinction coefficients of all the dyes were low independently from the coupling motif of the donor group to the acceptor heterocycle. The reason for low extinction coefficients is probably in the specific nature of [1,2,5]thiadiazolo[3,4-*d*]pyridazine, which is due to the electron deficient properties of the pyrydazine ring [33].

**Figure 4:**
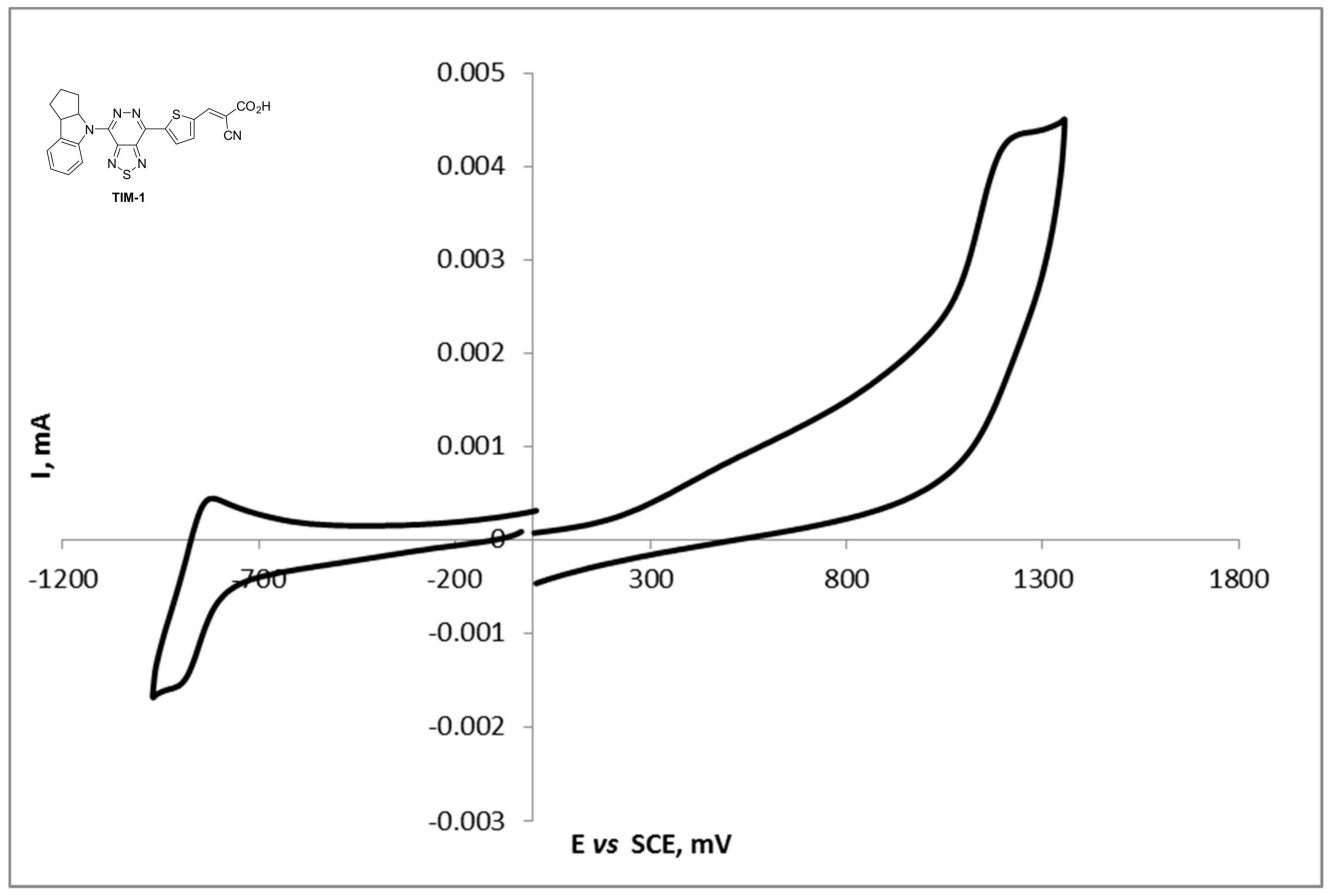

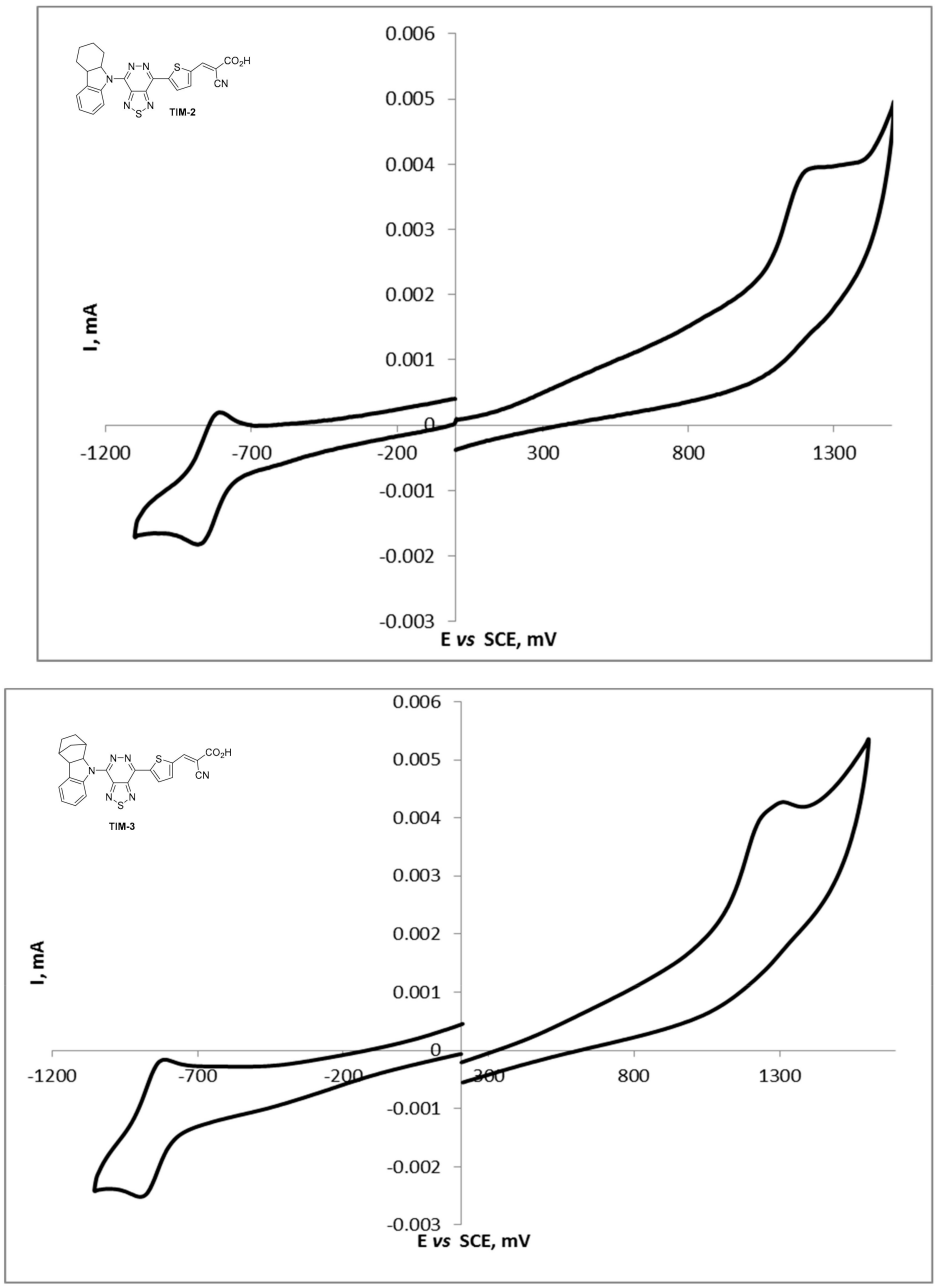

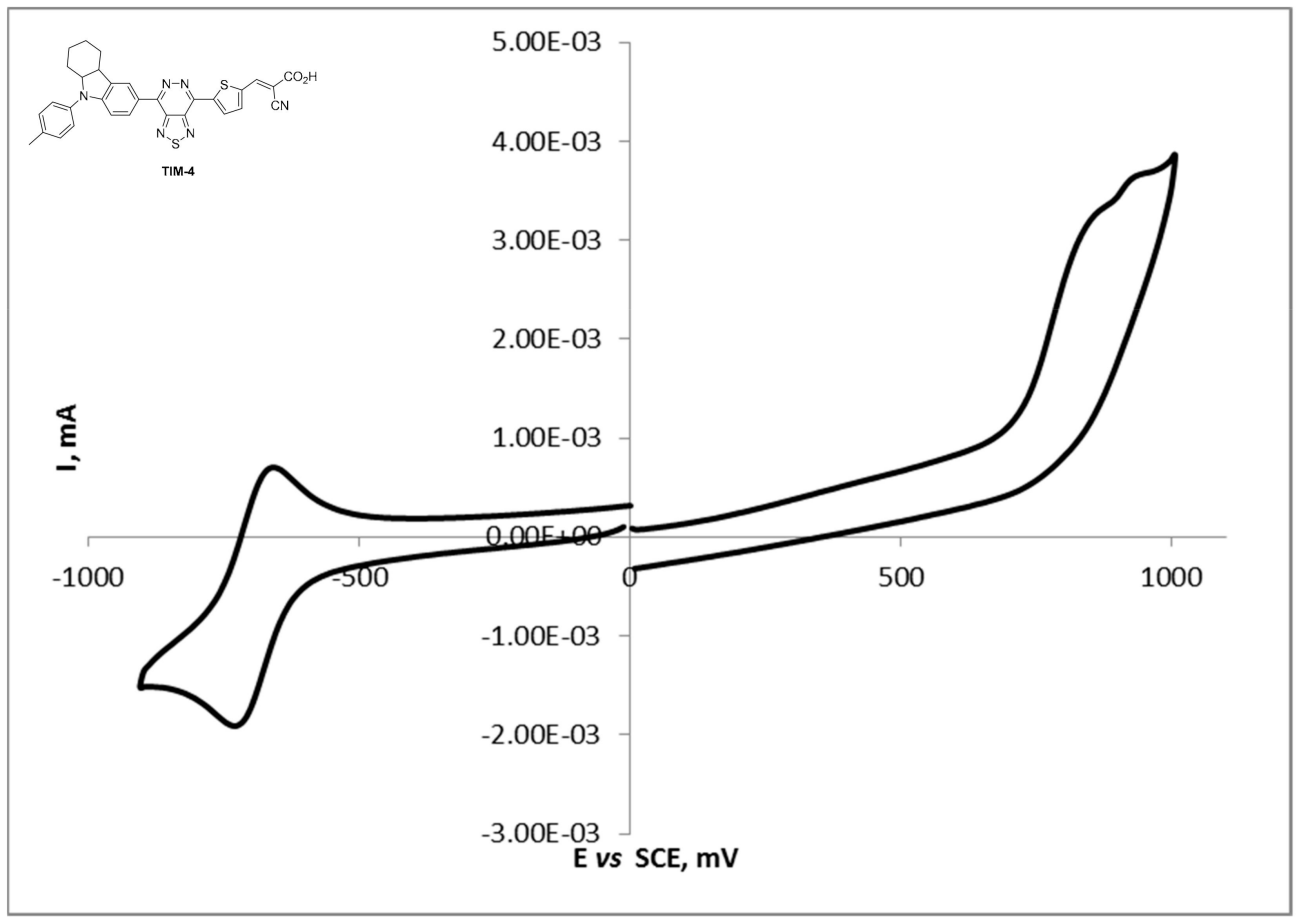
Cyclic voltammograms showing reduction and oxidation of **TIM1**–**4**. Scan rate 100 mVs^−1^, electrolyte 0.1 M Bu_4_NClO_4_ in DMF.

To estimate the energies of the frontier orbitals of the compounds studied, cyclic voltammograms of **TIM1–4** on a Pt electrode in DMF were obtained (Figure 4). **TIM1–4** electro-oxidation (EO) is irreversible even at high potential scan rate (10 Vs^−1^). The first stage of electric recovery (ER) of dyes **TIM1–4** is quasi-reversible at low values of the potential sweep rate (100 ms^−1^), and the cathode current ER is close to the current of a single-electron electrochemical reaction of ferrocene oxidation. To estimate the energy of the frontier orbitals, the potentials of the first stages of ER and EO **TIM1–4** were measured with respect to the internal standard, the reversible pair ferrocene/ferrocenium (Fc/Fc^+^), the absolute potential of which was taken as -5.1 eV [36]. The measured redox potentials are collected in Table 2. ER **TIM1–3** potentials had similar values (−1.28, −1.26, and −1.27 V, respectively) that is, **TIM1–3** substituents probably contributed almost the same to the energy of the E_LUMO_. The ER **TIM4** potential (−1.10 V) was 260–280 mV more positive than in the case of **TIM1–3**, which can be explained by an increase in conjugation in the **TIM4**.

Since the oxidation is irreversible and the formal potential was not determined, the values E_LUMO_ and E_HOMO_ were calculated by values of the potential onset of peaks ER ($E_{\mathrm{onset}}^{\mathrm{red}}$) and EO ($E_{\mathrm{onset}}^{\mathrm{ox}}$) (Table 2) according to the Equations (1) and (2) [36]:

(1) $$E_{\mathrm{HOMO}}\;(\mathrm{eV})\;=\;-\;|e|({E_{\mathrm{onset}}^{\mathrm{ox}}}_{,\;\mathrm{Fc}/\mathrm{Fc}+}\;+\;5.1)$$

(2) $$E_{\mathrm{LUMO}}\;(\mathrm{eV})\;=\;-\;|e|({E_{\mathrm{onset}}^{\mathrm{red}}}_{,\;\mathrm{Fc}/\mathrm{Fc}+}\;+\;5.1)$$

The values of E_LUMO_ of all studied compounds were more positive than the energy level of TiO_2_ semiconductor (−4.2 eV) [37], and all values of E_HOMO_ were more negative than the level of I^−^/I_3_^−^ (−5.2 eV) [38]. That is, on formal grounds, they meet the thermodynamic requirements for dyes. Moreover, the resulting compounds are characterized by a relatively narrow HOMO-LUMO gap $E_{\mathrm{gap}}^{\mathrm{CV}}$: **TIM1** (−1.82 eV) > **TIM2** (−1.80 eV) > **TIM3** (−1.82 eV) > **TIM4** (−1.31 eV), which, according to the literature [39], should enable good efficiency of solar cells.

The analysis of electrochemical parameters of dyes, which showed high efficiency values, indicated that the excess of the E_LUMO_ level over the energy level of TiO_2_ should be not less than 0.10–0.15 eV [40]. With regard to the efficiency of the regeneration of the dye, it is determined by the magnitude of E_HOMO_ compared to the level of I^−^/I^3−^ In different studies, this value has been considered to be optimal from 0.25 [41] to 0.75 eV [42]. Among the synthesized dyes **TIM1**–**4,** only the E_HOMO_ (−5.31 eV) for **TIM4** may not have a negative enough value.

The $E_{\mathrm{gap}}^{\mathrm{CV}}$ values obtained from the absorption onset of the **TIM** series dyes are consistent with the data obtained using CV. The **TIM4** compound, containing a C–C bond between the donor and the internal acceptor, has the lowest $E_{\mathrm{gap}}^{\mathrm{CV}}$ value compared to the three other dyes **TIM1–3** (−1.31 eV and −1.79 eV, −1.82 eV, respectively). Such a significant decrease in energy between HOMO and LUMO is probably due to an increase in the conjugation chain in the **TIM4** compound as compared to derivatives with C–N bonds, which is a consequence of the introduction of the *p*-tolyl substituent into the donor fragment.

### 2.3. DSSC Performance

**Figure 5:**
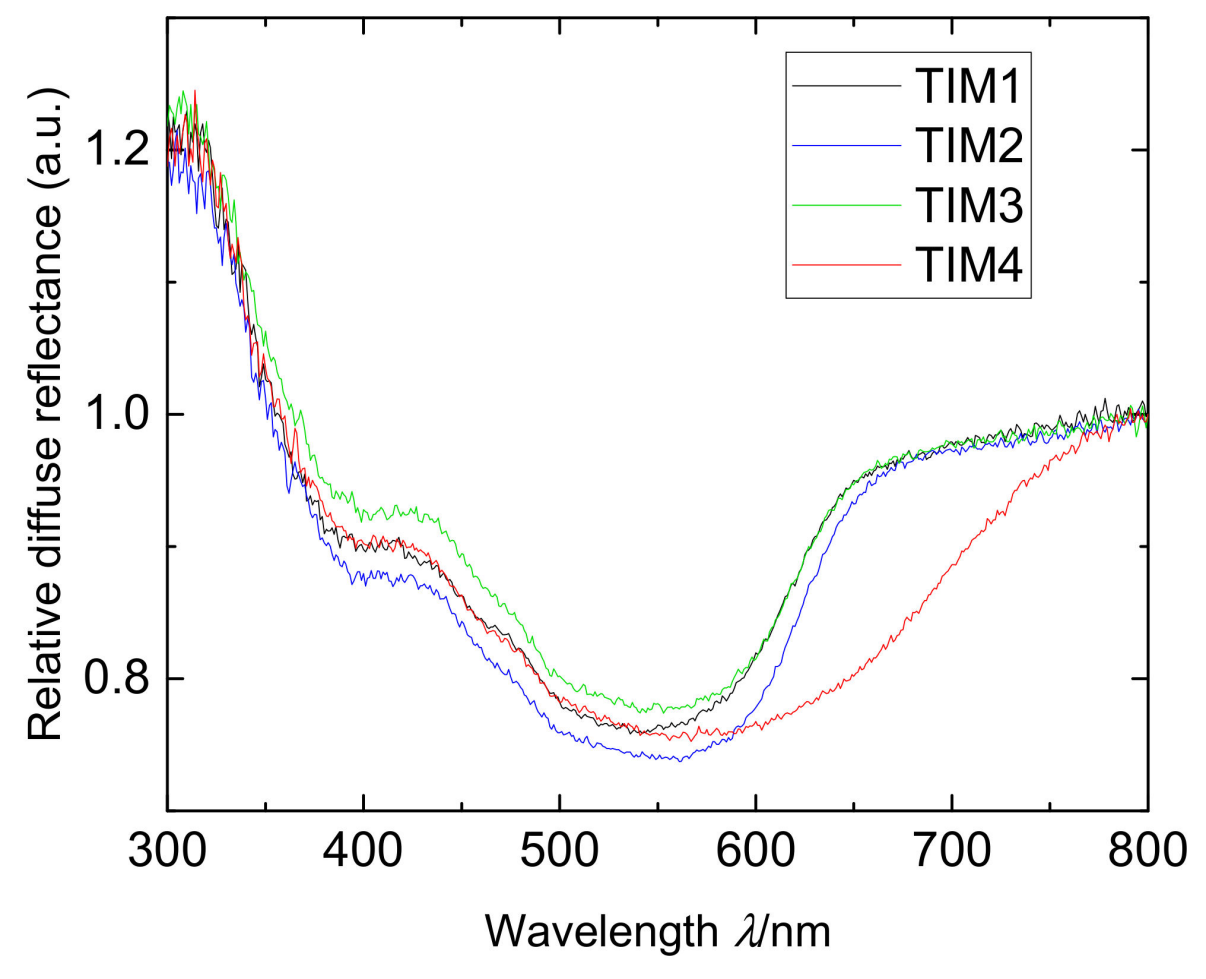
Diffuse reflectance of **TIM1**–**4**-coated TiO_2_ photoanodes. The light was irradiated from the non-FTO side. The irradiated area was fixed with a metal aperture to 0.0625 cm^2^ and an alumina block was placed at the back of the sample to exclude any transmittance. The *y*-axis shows the relative diffuse reflectance, by shifting the tail of the longer-wavelength peak (800 nm) to 1.

To evaluate the dye adsorption on the TiO_2_ film, diffuse reflectance was measured for the sensitized photoanodes prior to assembling the device. All samples were irradiated from the non-FTO side to simulate the actual device operation. In order to avoid transmittance effects and purely investigate the absorption, the reference alumina block was placed behind each sample during the measurement. In principle, the lower values in diffuse reflectance relate to higher absorption. The recorded spectra in Figure 5 show a clear drop in the diffuse reflectance around 400 nm and 550 nm, which indicates maximum light absorption of this spectral range by each dye. Similar to the trend in Figure 3, **TIM1**–**3** display a similar spectral profile, while **TIM4** shows a broader absorption from 600–800 nm, extending to the near-IR. For **TIM1**–**3,** the diffuse reflectance value of the longer-wavelength peak increased in the order of **TIM2**, **TIM1**, **TIM3**, which is in good accordance with the absorption profile of the solutions. These data indicate that the sensitization was performed in an adequate manner without any unwanted dye aggregation. The results from Figure 3 and Figure 5 imply that the best dye performance is expected for **TIM2**, in terms of light harvesting.

**Figure 6:**
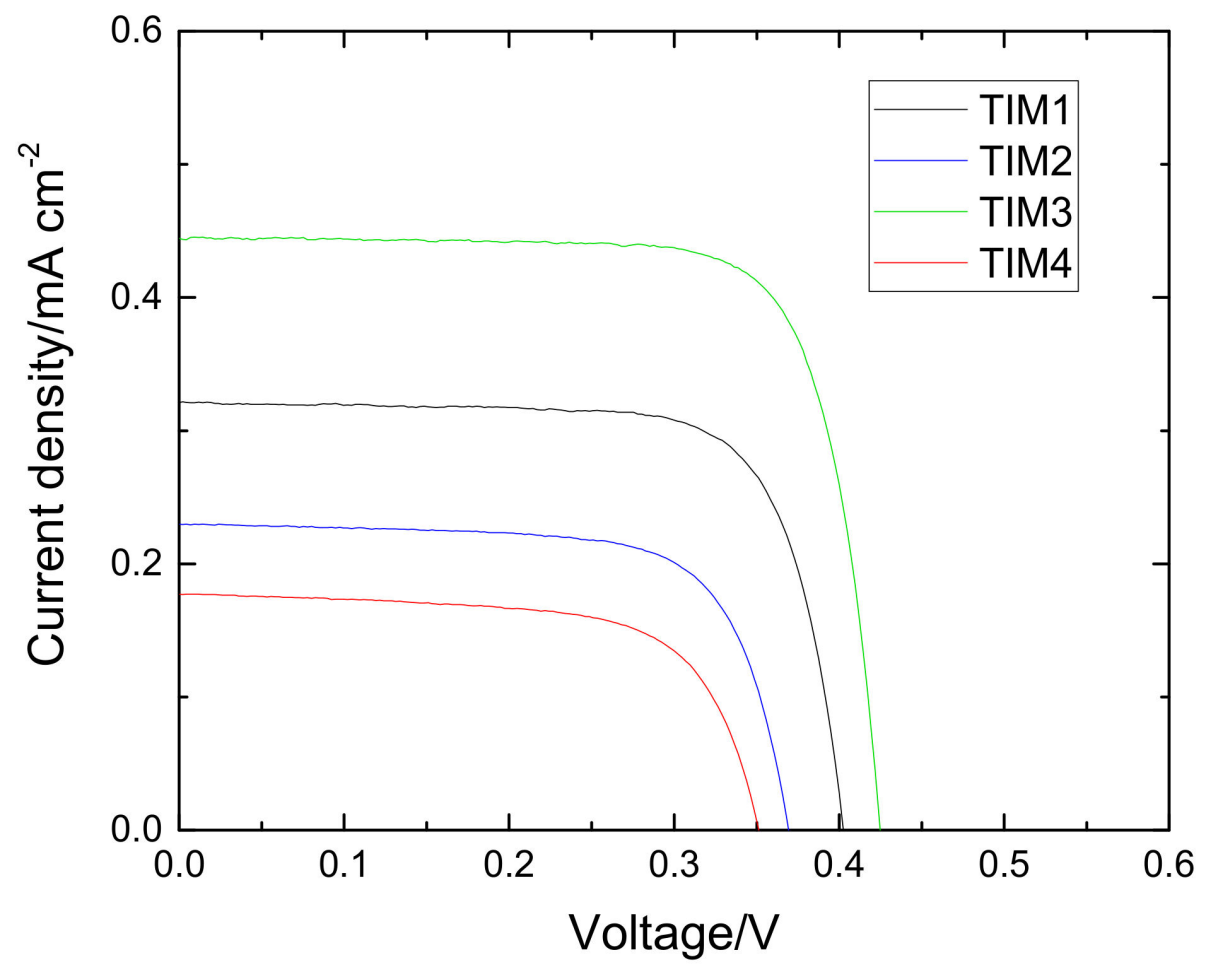
*J-V* curves of the dye-sensitized solar cells using **TIM1**–**4** series as the light absorber.

The photovoltaic characteristics of each dye in the assembled DSSCs are listed in Figure 6 and Table 3. The overall performance was low, probably due to the low extinction coefficients derived from Figure 3. Comparisons within the series however, may give pointers towards future dye design and performance. Firstly, **TIM4** showed the poorest performance, which is probably due to the *E*_HOMO_ mismatch suggested earlier. For **TIM1**–**3**, interestingly, both the current and voltage improved in the order of **TIM2** < **TIM1** < **TIM3**.

The outcome that **TIM3**, with the lowest light harvesting ability, exhibited the highest *J*_SC_ implies that the electron donating properties of the derivatives may be different. In addition, it is noteworthy that a distinct voltage difference was observed between **TIM1**–**3,** since the *E*_HOMO_ levels were shown to be identical for these dyes. The reasons behind the poorer results for **TIM1** and **TIM2** may be attributed to poor electron injection from the dye to the TiO_2_ and high charge recombination rate.

## 3. Experimental Section

### 3.1. Materials and Reagents

The reagents were purchased from commercial sources and used as received. 4,7-Dibromo[1,2,5]thiadiazolo[3,4-*d*]pyridazine (**1**) [34], 4-bromo-7-(1,3,3a,8b-tetrahydrocyclopenta[*b*]indol-4(2*H*)-yl)[1,2,5]thiadiazolo[3,4-*d*]pyridazine (**3a**) [34], 4-bromo-7-(2,3,4,4a-hexahydro-1*H*-carbazol-9 (9a*H*)-yl)[1,2,5]thiadiazolo[3,4-*d*]pyridazine (**3b**) [34], 4-bromo-7-(2,3,4,4a-tetrahydro-1*H*-1,4-methanocarbazol-9(9a*H*)-yl)[1,2,5]thiadiazolo[3,4-*d*]pyridazine(**3c**) [34], 4-bromo-7-[9-(p-*tolyl*)-2,3,4,4a,9,9a-hexahydro-1*H*-carbazol-6-yl][1,2,5]thiadiazolo[3,4-*d*]pyridazine (**4**) [33], *tert*-butyl 2-cyano-3-(4-(4,4,5,5-tetramethyl-1, and 3,2-dioxaboran-2-yl)thiophenyl-yl)acrylate (**5**) [43] were prepared according to the published methods and characterized by NMR spectra. All synthetic operations were performed under a dry argon atmosphere. Solvents were purified by distillation from the appropriate drying agents.

### 3.2. Analytical Instruments

Melting points were determined on a Kofler hot-stage apparatus and are uncorrected. ^1^H- and ^13^C-NMR spectra were taken with a Bruker AM-300 machine (at frequencies of 300.1 and 75.5 MHz, respectively) in CDCl_3_ solutions, with TMS as the standard. *J* values are given in Hz. MS spectra (EI, 70 eV) were obtained with a Finnigan MAT INCOS 50 instrument. High-resolution MS spectra were measured on a Bruker micrOTOF II instrument using electrospray ionization (ESI). The measurement was operated in a positive ion mode (interface capillary voltage −4500 V) or in a negative ion mode (3200 V); mass range was from *m*/*z* 50 to *m*/*z* 3000 Da; external or internal calibration was done with Electrospray Calibrant Solution (Fluka Chemicals Ltd., Gillingham, UK). A syringe injection was used for solutions in acetonitrile, methanol, or water (flow rate 3 μL·min^−1^). Nitrogen was applied as a dry gas; interface temperature was set at 180 °C. IR spectra were measured with a Bruker “Alpha-T” instrument (Bruker, Billerica, MA, USA) in KBr pellets.

### 3.3. General Procedure for Fabrication and Characterization of DSSCs

Fluorine doped tin oxide (FTO) conductive glass (2 mm thick, 7 Ω sheet resistance, Solaronix, Aubonne, Switzerland) was cleaned by sonication in 2% Decon 90 (Decon Laboratories, Hove, UK) in water, deionized water, acetone, and ethanol and treated with UV/O_3_ for 20 min. The substrates were treated with 40 mM TiCl_4_ at 80 °C for 30 min to form the blocking TiO_2_ layer. TiO_2_ paste 18NR-T and 18NR-AO (Dyesol, Queanbeyan, Australia) were deposited onto the blocking layer by screen printing, to obtain a film thickness of c.a. 7 µm (3 µm 18NR-T transparent layer +4 µm 18NR-AO scattering layer). The TiO_2_ anodes (area = 0.2728 cm^2^) were annealed in a furnace at 325 °C for 5 min, 375 °C for 5 min, 450 °C for 15 min, and finally 500 °C for 15 min (ramp 10 °C/min). Upon cooling to r.t., the treatment with 40 mM TiCl_4_ was repeated and the films were annealed at 500 °C for 30 min. The anodes were kept a 150 °C to release any trapped moisture before immersing in a 0.5 mM ethanol solution of **TIM** dyes with 5 mM chenodeoxycholic acid (CDCA) as an additive. Devices employing **N719** dye were also prepared for comparison. After an overnight sensitization, the anodes were rinsed with ethanol and dried in air. FTO glass for the cathodes was cleaned by sonication in 0.1 M HCl in ethanol, deionized water, acetone, and ethanol. The substrates were platinized by doctor-blading Platisol T/SP (Solaronix, Aubonne, Switzerland) and annealed at 450 °C for 10 min. The solar cells were assembled by binding the two electrodes together with a piece of Surlyn (25 µm, Du Pont, Wilmington, USA) at 125 °C. Electrolyte was vacuum filled into a pre-drilled hole and the hole was sealed by Surlyn and a cover glass. The electrolyte was composed of 0.1 M LiI, 0.05 M I_2_, 0.6 M 1,2-dimethyl-3-propylimidazolium iodide (DMPII), and 0.5 M 4-*tert*-butylpyridine in anhyd. acetonitrile.

Current–voltage (*I*–*V*) curves were measured with a potentiostat (Metrohm, Autolab, Herisau, Switzerland) and a class AAA solar simulator (SLB300A, Sciencetech, London, Ontario, Canada). The intensity of the incident light was calibrated to AM1.5G sunlight (100 mW cm^−2^) using a Si reference cell. The solar cells were masked with a metal aperture to define the active area to 0.0625 cm^2^.

### 3.4. Detailed Experimental Procedures and Characterization Data

#### 3.4.1. Optical Characterization

Solution UV–visible absorption spectra were recorded using a Jasco V-670 UV/Vis/NIR spectrophotometer (JASCO, Mary’s Court Easton, MD, USA) controlled with SpectraManager software. All samples were measured in a 1 cm quartz cell at room temperature with 5 × 10^−5^ mol mL^−1^ concentration in EtOH.

#### 3.4.2. Electrochemical Characterization

Electrochemical measurements were carried out in a dry argon atmosphere using an IPC Pro MF potentiostat. The redox properties of compounds were determined using cyclic voltammetry in a three-electrode electrochemical system. A three-electrode system consisting of platinum as working electrode with an area of 0.8 mm^2^, platinum wire as counter electrode, and saturated calomel electrode (SCE) as reference electrode was employed. The reduction and oxidation potentials were determined in DMF, using 0.1 mol L^−1^ n-Bu_4_NClO_4_ as the supporting electrolyte. Cyclic voltammetry (CV) measurements used scan rates of 0.1 V·s^−1^. The first reduction/oxidation potentials were referenced to the internal standard redox couple Fc/Fc^+^. Ferrocene was added to each sample solution at the end of the experiment, and employed for calibration.

#### 3.4.3. Synthesis and Characterization of Compounds

tert-Butyl 2-cyano-3-(5-(tributylstannyl)thiophen-2-yl)acrylate (**6**): A mixture of *tert*-butyl 2-cyano-3-(4-(4,4,5,5-tetramethyl-1,3,2-dioxaboran-2-yl)thiophenyl-yl) acrylate **5** (0.3 mmol) and Bu_3_SnOMe (0.3 mmol) was heated at 110 °C in an argon atmosphere for = 10 h in a sealed vessel. After completion of the chemical reaction (monitored by TLC), the mixture was purified by silica gel column chromatography (eluent: CH_2_Cl_2_/hexane, 1:2). Colorless oil, yield 63 mg (40%), R_f_ = 0.6 (CH_2_Cl_2_/ petroleum ether = 1:1). IR *ν*_max_ (KBr, cm^−1^): 2958, 2930, 2853, 2218, 1717, 1590, 1457, 1407, 1370, 1300, 1280, 1238, 1159, 1060, 939, 841, 752, 668, 503. ^1^H NMR (300 MHz, CDCl_3_): 0.92 (t, *J* = 7.2 Hz, 9H), 1.16–1.21 (m, 5Н), 1.30–1.42 (m, 7H), 1.54–1.62 (m, 15H), 7.28 (d, *J* = 3.4 Hz, 1H), 7.90 (d, *J* = 3.4 Hz, 1H), 8.27 (s, 1H). ^13^C- NMR (75 MHz, CDCl_3_): *δ* 11.2, 13.6, 27.2, 28.1, 28.9, 83.3, 99.9, 116.4, 136.6, 136.8, 141.5, 144.9, 152.2, 162.1. HRMS (ESI- TOF), *m*/*z*: calcd for C_24_H_39_NO_2_S^120^SnNa [M + Na]^+^, 548.1618, found 548.1624.

##### General procedure for the of cross-coupling reaction of mono-adducts **3**(**a**–**c**), **4**, and stannane **6**.

To a solution of mono-substituted products **3a**–**c**, **4** (0.25 mmol) in anhydrous THF (5 mL) were added PdCl_2_(PPh_3_)_2_ (3% mol) and stannane **6** (0.3 mmol). The resulting cloudy yellow mixture was stirred and degassed by argon and refluxed under argon for 8 h. After cooling, an additional amount of stannane **6** (0.3 mmol) and PdCl_2_(PPh_3_)_2_ (3% mol) were added and the reaction mixture was refluxed for 8 h. On completion (monitored by TLC), the mixture was washed with water and the organic layer was extracted with CH_2_Cl_2_ (3 × 50 mL). The combined organic layers were washed with brine, dried over MgSO_4_ and concentrated under reduced pressure. The crude product was purified by column chromatography.

*tert-Butyl 2-cyano-3-(5-(7-(1,3,3a,8b-tetrahydrocyclopenta[b]indol-4(2H)-yl)-[1,2,5]thiadiazolo[3,4-d]pyridazin-4-yl)thiophen-2-yl)acrylate* (**7a**): Dark red solid, yield 108 mg (85%), R_f_ = 0.3 (CH_2_Cl_2_). Mp 89–91 °C. Eluent—CH_2_Cl_2_:hexane, 1:1 (*v*/*v*). IR *ν*_max_ (KBr, cm^−1^): 2955, 2931, 2865, 2216, 1713, 1585, 1503, 1457, 1424, 1367, 1287, 1255, 1244, 1211, 1152, 1109, 1051, 840, 752, 521. ^1^H-NMR (300 MHz, CDCl_3_): *δ* 1.44–1.52 (m, 1H), 1.62 (s, 9H), 1.68–1.81 (m, 2H), 2.05–2.17 (m, 2H), 2.20–2.35 (m, 1H), 4.05–4.10 (m, 1H), 5.97 – 6.02 (m, 1H), 7.12 (t, *J* = 7.3 Hz, 1H), 7.23 (d, *J* = 7.3 Hz, 1H), 7.30 (t, *J* = 8.4 Hz, 1H), 7.93 (d, *J* = 4.1 Hz, 1H), 8.22 (s, 1H), 8.47 (d, *J* = 4.1 Hz, 1H), 8.87 (d, *J* = 8.4 Hz, 1H). ^13^C-NMR (75 MHz, CDCl_3_): *δ* 23.8, 28.0, 34.1, 36.7, 45.8, 67.9, 83.5, 101.2, 115.9, 119.2, 124.0, 124.7, 127.7, 129.4, 136.4, 136.7, 136.9, 142.3, 143.3, 143.4, 145.1, 147.3, 149.3, 149.4, 161.6. HRMS (ESI-TOF), *m*/*z*: calcd for C_27_H_25_N_6_O_2_S_2_ [M + H]^+^, 529.1475, found 529.1473.

*tert-Butyl 2-cyano-3-(5-(7-(2,3,4,4a-tetrahydro-1H-carbazol-9(9aH)-yl)-[1,2,5]thiadiazolo[3,4-d]pyridazin-4-yl)thiophen-2-yl)acrylate* (**7b**): Dark red solid, yield 105 mg (80%), R_f_ = 0.3 (CH_2_Cl_2_). Mp 199–201 °C. Eluent—CH_2_Cl_2_:hexane, 1:1 (*v*/*v*). IR *ν*_max_ (KBr, cm^−1^): 2925, 2854, 2216, 1701, 1593, 1506, 1457, 1425, 1369, 1290, 1268, 1258, 1233, 1210, 1152, 1092, 1057, 1018, 910, 837, 826, 757, 722, 522, 425. ^1^H-NMR (300 MHz, CDCl_3_): *δ* 1.33–1.43 (m, 3H), 1.62 (s, 9H), 1.67–1.74 (m, 2H), 1.92–2.06 (m, 1H), 2.25–2.30 (m, 1H), 2.47 (d, *J* = 14.1 Hz, 1H), 3.71–3.75 (m, 1H), 5.85–5.90 (m, 1H), 7.21 (t, *J* = 7.3 Hz, 1H), 7.32 (d, *J* = 7.3 Hz, 1H), 7.38 (t, *J* = 8.0 Hz, 1H), 8.00 (d, *J* = 4.0 Hz, 1H), 8.26 (s, 1H), 8.54 (d, *J* = 4.0 Hz, 1H), 8.78 (d, *J* = 8.0 Hz, 1H). ^13^C-NMR (75 MHz, CDCl_3_): *δ* 20.9, 22.9, 24.3, 28.1, 29.8, 40.3, 64.0, 83.6, 101.2, 116.1, 120.7, 122.6, 124.7, 127.5, 129.4, 135.4, 136.8, 137.0, 142.5, 142.6, 143.0, 145.3, 147.5, 149.3, 149.5, 161.8. HRMS (ESI-TOF), *m*/*z*: calcd for C_28_H_27_N_6_O_2_S_2_ [M + H]^+^, 543.1631, found 543.1622.

*tert-Butyl 2-cyano-3-(5-(7-(2,3,4,4a-tetrahydro-1H-1,4-methanocarbazol-9(9aH)-yl)-[1,2,5]thiadiazolo[3,4-d]pyridazin-4-yl)thiophen-2-yl)acrylate* (**7c**): Dark red solid, yield 103 mg (75%), R_f_ = 0.3 (CH_2_Cl_2_). Mp 218–220 °C. Eluent—CH_2_Cl_2_:hexane, 1:1 (*v*/*v*). IR *ν*_max_ (KBr, cm^−1^): 2956, 2924, 2853, 2216, 1716, 1586, 1502, 1457, 1423, 1368, 1292, 1284, 1250, 1211, 1153, 1108, 1037, 813, 752, 522. ^1^H-NMR (300 MHz, CDCl_3_): 1.10 (d, *J* = 10.7 Hz, 1H), 1.39 (d, *J* = 10.06 Hz, 1H), 1.54–1.57 (m, 1H), 1.62 (s, 9H), 1.64–1.72 (m, 3H), 2.43 (d, *J* = 20.0 Hz, 2H), 3.53 (d, *J* = 7.8 Hz, 1H), 5.45 (d, *J* = 7.8 Hz, 1H), 7.11 (t, *J* = 7.3 Hz, 1H), 7.23 (d, *J* = 7.3 Hz, 1H), 7.30 (t, *J* = 8.2 Hz, 1H), 7.94 (d, *J* = 4.2 Hz, 1H), 8.23 (s, 1H), 8.49 (d, *J* = 4.2 Hz, 1H), 8.90 (d, *J* = 8.2 Hz, 1H). ^13^C-NMR (75 MHz, CDCl_3_): *δ* 25.9, 28.0, 28.1, 31.9, 43.6, 43.7, 50.6, 69.8, 83.6, 101.3, 116.0, 119.0, 124.3, 124.5, 127.9, 129.5, 135.5, 136.7, 137.1, 142.5, 143.4, 144.9, 145.2, 147.4, 149.4, 149.7, 161.7. HRMS (ESI-TOF), *m*/*z*: calcd for C_29_H_27_N_6_O_2_S_2_ [M + H]^+^, 555.1620, found 555.1631.

*tert-Butyl 2-cyano-3-(5-(7-(9-(p-tolyl)-2,3,4,4a,9,9a-hexahydro-1H-carbazol-6-yl)-[1,2,5]thiadiazolo[3,4-d]pyridazin-4-yl)thiophen-2-yl)acrylate* (**7d**): Violet-green solid, yield 110 mg (70%), R_f_ = 0.3 (CH_2_Cl_2_). Mp 110–112 °C. Eluent—CH_2_Cl_2_:hexane, 1:1 (*v*/*v*). IR *ν*_max_ (KBr, cm^−1^): 2924, 2853, 2217, 1718, 1603, 1587, 1513, 1474, 1452, 1399, 1270, 1246, 1154, 812, 523. ^1^H-NMR (300 MHz, CDCl_3_): *δ* 1.40–1.56 (m, 4H), 1.62 (s, 9H), 1.76–1.77 (m, 2H), 1.92–2.00 (m, 2H), 2.40 (s, 3H), 3.38–3.45 (m, 1H), 4.23–4.29 (m, 1H), 6.87 (d, *J* = 8.5 Hz, 1H), 7.21–7.28 (m, 4H), 8.01 (d, *J* = 4.2 Hz, 1H), 8.27 (s, 1H), 8.66 (d, *J* = 1.5 Hz, 1H), 8.69 (d, *J* = 4.2 Hz, 1H), 8.72 (d, *J* = 1.5 Hz, 1H). ^13^C-NMR (75 MHz, CDCl_3_): *δ* 21.0, 21.1, 22.3, 26.0, 27.7, 28.0, 40.2, 65.1, 83.8, 102.5, 108.5, 115.8, 123.4, 124.0, 124.8, 130.1, 131.7, 131.8, 134.4, 135.6, 136.5, 139.0, 139.1, 144.9, 146.2, 146.4, 148.5, 149.1, 152.6, 152.8, 161.4. HRMS (ESI-TOF), *m*/*z*: calcd for C_35_H_33_N_6_O_2_S_2_ [M + H]^+^, 633.2101, found 633.2084.

##### General procedure for hydrolysis of ethers **7(a**–**g)**.

To the solution of ether **7a**–**d** (0.2 mmol) in chloroform (10 mL), trifluoroacetic acid (4 mmol) was added and the mixture was refluxed for 8 h. After cooling, the mixture was concentrated under residue pressure. The crude product was purified by column chromatography.

*2-Cyano-3-(5-(7-(1,3,3a,8b-tetrahydrocyclopenta[b]indol-4(2H)-yl)-[1,2,5]thiadiazolo[3,4-d]pyridazin-4-yl)thiophen-2-yl)acrylic acid* (**TIM-1**): Dark red solid, yield 85 mg (90%), R_f_ = 0.1 (EtOAc). Mp 253–255 °C. Eluent—MeOH:EtOAc, 1:1 (*v*/*v*). IR *ν*_max_ (KBr, cm^−1^): 2959, 2928, 2215, 1685, 1576, 1522, 1508, 1425, 1363, 1293, 1262, 1211, 1140, 1051, 1021, 755, 665, 521. ^1^H-NMR (300 MHz, DMSO-d_6_): *δ* 1.29–1.34 (m, 1H), 1.65–1.69 (m, 1H), 1.98–2.03 (m, 1H), 2.10–2.50 (m, 3H), 4.08–4.12 (m, 1H), 5.96–6.01 (m, 1H), 7.12 (t, *J* = 7.2 Hz, 1H), 7.29 (t, *J* = 8.1 Hz, 1H), 7.34 (d, *J* = 7.2 Hz, 1H), 7.79 (d, *J* = 3.9 Hz, 1H), 8.13 (s, 1H), 8.42 (d, *J* = 3.9 Hz, 1H), 8.77 (d, *J* = 8.1 Hz, 1H). ^13^C-NMR (75 MHz, DMSO-d_6_): *δ* 23.2, 33.5, 35.9, 44.9, 67.2, 111.5, 117.8, 118.8, 123.7, 123.9, 127.0, 128.6, 134.5, 136.1, 138.4, 139.3, 142.3, 142.5, 142.9, 143.2, 148.6, 149.1, 162.7. HRMS (EI-MS): calcd. for C_23_H_16_N_6_O_2_S_2_Na [M + Na]^+^, 495.0668, found 495.0668.

*2-Cyano-3-(5-(7-(2,3,4,4a-tetrahydro-1H-carbazol-9(9aH)-yl)-[1,2,5]thiadiazolo[3,4-d]pyridazin-4-yl)thiophen-2-yl)acrylic acid* (**TIM-2**): Dark red solid, yield 87 mg (90%), R_f_ = 0.1 (EtOAc). Mp 138–140 °C. Eluent—MeOH:EtOAc, 1:1 (*v*/*v*). IR *ν*_max_ (KBr, cm^−1^): 2927, 2855, 2215, 1680, 1510, 1459, 1380, 1295, 1212, 1152, 848, 803, 726, 520. ^1^H-NMR (300 MHz, DMSO-d_6_): *δ* 1.34–1.37 (m, 3H), 1.60–1.63 (m, 2H), 1.91–1.95 (m, 1H), 2.18–2.24 (m, 1H), 2.42 (d, *J* = 13.9 Hz, 1H), 3.71–3.73 (m, 1H), 5.80–5.85 (m, 1H), 7.18 (t, *J* = 7.2 Hz, 1H), 7.32 (t, *J* = 8.3 Hz, 1H), 7.39 (d, *J* = 7.2 Hz, 1H), 7.84 (d, *J* = 3.9 Hz, 1H), 8.20 (s, 1H), 8.43 (d, *J* = 3.9 Hz, 1H), 8.65 (d, *J* = 8.3 Hz, 1H). ^13^C-NMR (75 MHz, DMSO-d_6_): *δ* 20.3, 21.8, 23.3, 27.3, 40.4, 62.8, 109.9, 118.4, 121.5, 122.4, 123.7, 126.7, 128.6, 135.0, 135.2, 137.9, 140.3, 142.3, 142.4, 142.5, 143.1, 148.6, 149.0, 163.6. HRMS (EI-MS): calcd. for C_24_H_18_N_6_O_2_S_2_Na [M + Na]^+^, 509.0825, found 509.0815.

*2-Cyano-3-(5-(7-(1,2,3,4,4a,9a-hexahydro-9H-1,4-methanocarbazol-9-yl)-[1,2,5]thiadiazolo[3,4-d]pyridazin-4-yl)thiophen-2-yl)acrylic acid* (**TIM3**): Dark red solid, yield 85 mg (86%), R_f_ = 0.1 (EtOAc). Mp > 260 °C. Eluent—MeOH:EtOAc, 1:1 (*v*/*v*). IR *ν*_max_ (KBr, cm^−1^): 2929, 2856, 2216, 1686, 1576, 1507, 1423, 1211, 1189, 1140, 840, 660. ^1^H-NMR (300 MHz, DMSO-d_6_): δ 1.09 (d, *J* = 10.7 Hz, 1H), 1.17 (d, *J* = 10.06 Hz, 1H), 1.58–1.62 (m, 4H), 2.43–2.47 (m, 2H), 3.60 (d, *J* = 7.9 Hz, 1H), 5.49 (d, *J* = 7.9 Hz, 1H), 7.09 (t, *J* = 7.3 Hz, 1H), 7.27 (d, *J* = 7.3 Hz, 1H), 7.33 (t, *J* = 8.2 Hz, 1H), 7.78 (d, *J* = 4.0 Hz, 1H), 8.08 (s, 1H), 8.42 (d, *J* = 4.0 Hz, 1H), 8.78 (d, *J* = 8.2 Hz, 1H). ^13^C-NMR (75 MHz, DMSO-d_6_): *δ* 24.9, 27.3, 31.3, 42.8, 42.9, 49.6, 68.9, 111.9, 117.6, 119.2, 123.7, 124.4, 127.3, 128.7, 135.1, 135.2, 138.5, 139.3, 142.5, 142.6, 143.2, 144.7, 148.7, 149.4, 162.2. HRMS (EI-MS): calcd. for C_25_H_18_N_6_O_2_S_2_Na [M + Na]^+^, 521.0825, found 521.0820.

*2-Cyano-3-(5-(7-(9-(p-tolyl)-2,3,4,4a,9,9a-hexahydro-1H-carbazol-6-yl)-[1,2,5]thiadiazolo[3,4-d]pyridazin-4-yl)thiophen-2-yl)acrylic acid* (**TIM-4**): Violet solid, yield 98 mg (85%), R_f_ = 0.1 (EtOAc), Mp > 260 °C. Eluent—MeOH:EtOAc, 1:1 (*v*/*v*). IR *ν*_max_ (KBr, cm^−1^): 2926, 2854, 2213, 1718, 1603, 1561, 1513, 1450, 1399, 1376, 1272, 1135, 1108, 811, 522. ^1^H-NMR (300 MHz, DMSO-d_6_): *δ* 1.42–1.61 (m, 4H); 1.53–1.67 (m, 2H); 1.90–2.01 (m, 2H); 2.34 (s, 3H); 3.39–3.46 (m, 1H); 4.29–4.35 (m, 1H); 6.82 (d, *J* = 8.6 Hz, 1H); 7.22–7.29 (m, 4H); 7.83 (d, *J* = 4.1 Hz, 1H); 8.09 (s, 1H); 8.49–8.53 (m, 3H), 8.57 (d, *J* = 4.1 Hz, 1H). ^13^C-NMR (75 MHz, DMSO-d_6_): *δ* 20.3, 20.4, 21.7, 25.1, 27.2, 40.4, 63.8, 107.8, 119.1, 122.7, 124.0, 124.1, 126.5, 130.0, 130.5, 131.4, 133.3, 135.0, 135.1, 138.7, 139.1, 140.7, 141.6, 146.2, 147.7, 148.4, 151.2, 151.7, 162.2. HRMS (EI-MS): calcd. for C_31_H_24_N_6_O_2_S_2_Na [M + Na]^+^, 599.1294, found 599.1287.

## 4. Conclusions

In summary, we have designed four new D-A-π-A metal-free organic sensitizers with [1,2,5]thiadiazolo[3,4-*d*]pyridazine as the internal acceptor, thiophene unit as the π-spacer, and cyanoacrylate as the anchoring electron acceptor. Despite the variation of the donor fragment and manner of its attachment to the internal acceptor, low PCE data were measured for all dyes. There are a few conclusions that can be made from the results obtained. First of all, comparison of the PCE for **TIM1**–**TIM3** drives us to conclude that there is no significant impact originating from the *N*-donor; they showed quite similar PCE values, with a slightly better result for the 2,3,4,4a,9,9a-hexahydro-1*H*-1,4-methanocarbazolyl moiety (**TIM3**). These data are in good agreement with the data obtained by us earlier, with sensitizers based on 9-(*p*-tolyl)-2,3,4,4a,9,9a-hexahydro-1*H*-carbazole (used in **TIM4**) showing similar results to the well-known 4-(*p*-tolyl)-1,2,3,3a,4,8b-hexahydrocyclopenta[*b*]indolyl building block (see, for example, dye **WS-2** [4]). Secondly, the comparison of PCE data for **TIM2** and **TIM4** showed practically identical values, indicating a small difference in attachment of the donor fragment to the internal acceptor moiety, which may open another interesting possibility for flexibility of the chemical structures of the dyes. Finally, the relatively low performance suggests that the internal [1,2,5]thiadiazolo[3,4-*d*]pyridazine acceptor was responsible for poor PCE data due to lower light harvesting by the dye, which is not beneficial when designing D-A-π-A dyes for DSSCs. This is not surprising, since benzo[*c*][1,2,5]thiadiazoles showed much better PCE values than [1,2,5]thiadiazolo[3,4-*c*]pyridines; apparently, employing high electron-accepting internal building blocks is unfavorable to the improvement of PCE.

## Figures and Tables

**Table 1:** The absorption peaks (λ_max_) and their corresponding molar absorption coefficients (ε) of the **TIM** series.

Dye	λ_max1_ [nm] ^a^	ε_max1_ × 10^3^ [M^−1^·cm^−1^] ^a^	λ_max2_ [nm]^a^	ε_max2_ × 10^3^ [M^−1^·cm^−1^] ^a^	λ_onset_ [nm] ^a^	$E_{\mathrm{gap}}^{\mathrm{opt}}\;[\mathrm{eV}]{\;}^{b}$
**TIM1**	383	2.7	524	2.3	631	1.968
**TIM2**	382	4.1	520	3.6	617	2.013
**TIM3**	382	1.8	526	1.7	620	2.003
**TIM4**	-	-	554	1.6	722	1.720

^a^ Absorption peaks (λ_max_) and molar extinction coefficients (ε_max_) in EtOH; ^b^ Calculated by 1.242/λ_onset._

**Table 2:** Electrochemical properties of the dyes **TIM1–4** in DMF solution.

Dye	E_ox_ [V] vs. Fc/Fc^+^^a^	E_red_ [V] vs Fc/Fc^+^^a^	Е_HOMO_ [eV] ^b^	Е_LUMO_ [eV] ^b^	$E_{\mathrm{gap}}^{\mathrm{CV}}\;[\mathrm{eV}{]}^{\;c}$
**TIM1**	0.54	−1.28	−5.64	−3.82	−1.82
**TIM2**	0.54	−1.26	−5.64	−3.84	−1.80
**TIM3**	0.52	−1.27	−5.62	−3.83	−1.79
**TIM4**	0.21	−1.10	−5.31	−4.00	−1.31

^a^ Here E_ox_ and E_red_ are a linear extrapolation of the low reduction potential side of the first oxidation or reduction wave respectively to the base line relative to Fc/Fc^+^, respectively; ^b^ Energies of frontier orbitals were calculated according to Equations (1) and (2); ^c^ $E_{\mathrm{gap}}^{\mathrm{CV}}$ = Е_LUMO_ − Е_HOMO._

**Table 3:** Summary of the device performance for the champion cells. The data for **N719** dye are shown as a reference.

Dye	*V*_OC_ [V]	*J*_SC_ [mA cm^−2^]	FF	PCE [%]
**N719**	0.67	16.35	0.76	8.29
**TIM1**	0.39	0.32	0.75	0.09
**TIM2**	0.37	0.23	0.71	0.06
**TIM3**	0.42	0.45	0.76	0.14
**TIM4**	0.35	0.20	0.68	0.05

## Supplementary Materials

Results of quantum calculations and characterization data including ^1^H and ^13^C NMR spectra for the compounds **6**, **7a**–**d**, **TIM1**–**3**.
