# Supplementary material for: [1,2,5]Thiadiazolo[3,4-d]Pyridazine as an Internal Acceptor in the D-A-π-A Organic Sensitizers for Dye-Sensitized Solar Cells
Source: Molecules. 2019 Apr 22;24(8):1588. doi: 10.3390/molecules24081588 (PMC6515329; doi:10.3390/molecules24081588)
Supplement: Supplementary file 1 [file molecules-24-01588-s001.pdf]

Supplementary Materials

[1,2,5]Thiadiazolo[3,4-*d*]Pyridazine as an Internal Acceptor in the D-A-π-A Organic Sensitizers for Dye-Sensitized Solar Cells

Timofey N. Chmovzh [1], Ekaterina A. Knyazeva [1,2], Ellie Tanaka [3], Vadim V. Popov [2], Ludmila V. Mikhalchenko [1], Neil Robertson [3,*] and Oleg A. Rakitin [1,2,*]

[1] N. D. Zelinsky Institute of Organic Chemistry, Russian Academy of Sciences, 119991 Moscow, Russia; orakitin@ioc.ac.ru (O.A.R.); tim1661@yandex.ru (T.N.C.); katerina_knyazev@ioc.ac.ru (E.A.K.); mlv@ioc.ac.ru (L.V.M.)

[2] Nanotechnology Education and Research Center, South Ural State University, 454080 Chelyabinsk, Russia; popov.ioc@gmail.com

[3] EaStCHEM School of Chemistry, University of Edinburgh, Edinburgh EH9 3FJ, UK; Ellie.Tanaka@ed.ac.uk

* Correspondence: Correspondence: Neil.Robertson@ed.ac.uk (N.R.); orakitin@ioc.ac.ru (O.A.R.); Tel.: +44 131 6504755 (N.R.); +7-499-135-5327(O.A.R.)

1. ^{1}H and ^{13}C NMR spectra……………………………………………………………2-10

2. Quantum-chemical calculations………………………………………………………11-24

1. ¹H and ¹³C NMR spectra

tert-Butyl 2-cyano-3-(5-(tributylstannyl)thiophen-2-yl)acrylate (6)

¹H NMR(300 MHz)

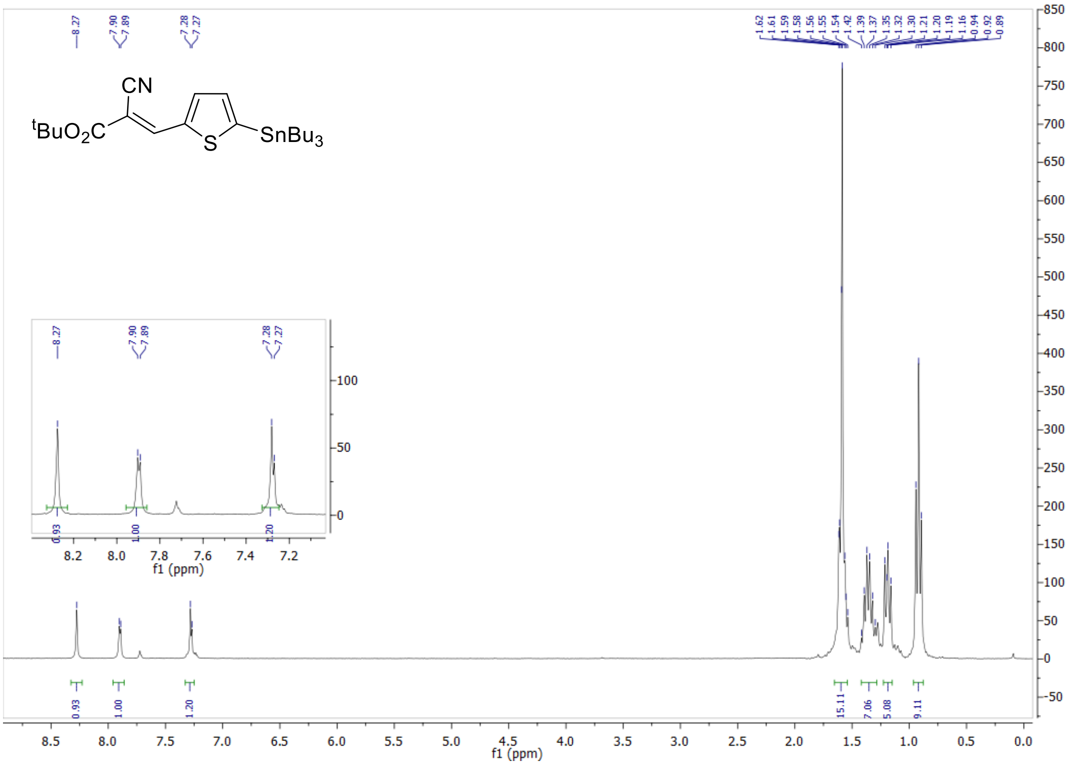

¹³C NMR(75 MHz)

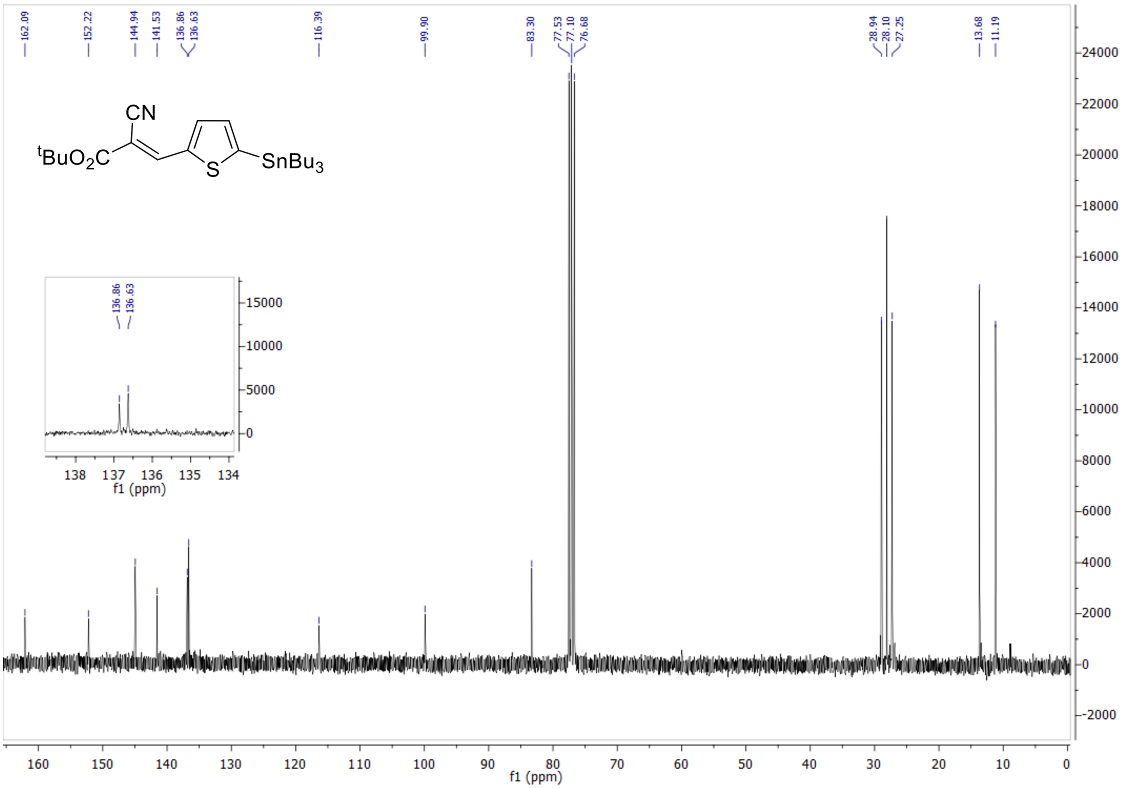

tert-Butyl 2-cyano-3-(5-(7-(1,3,3a,8b-tetrahydrocyclopenta[b]indol-4(2H)-yl)-[1,2,5]thiadiazolo[3,4-d]pyridazin-4-yl)thiophen-2-yl)acrylate (7a)

^{1}H NMR(300 MHz)

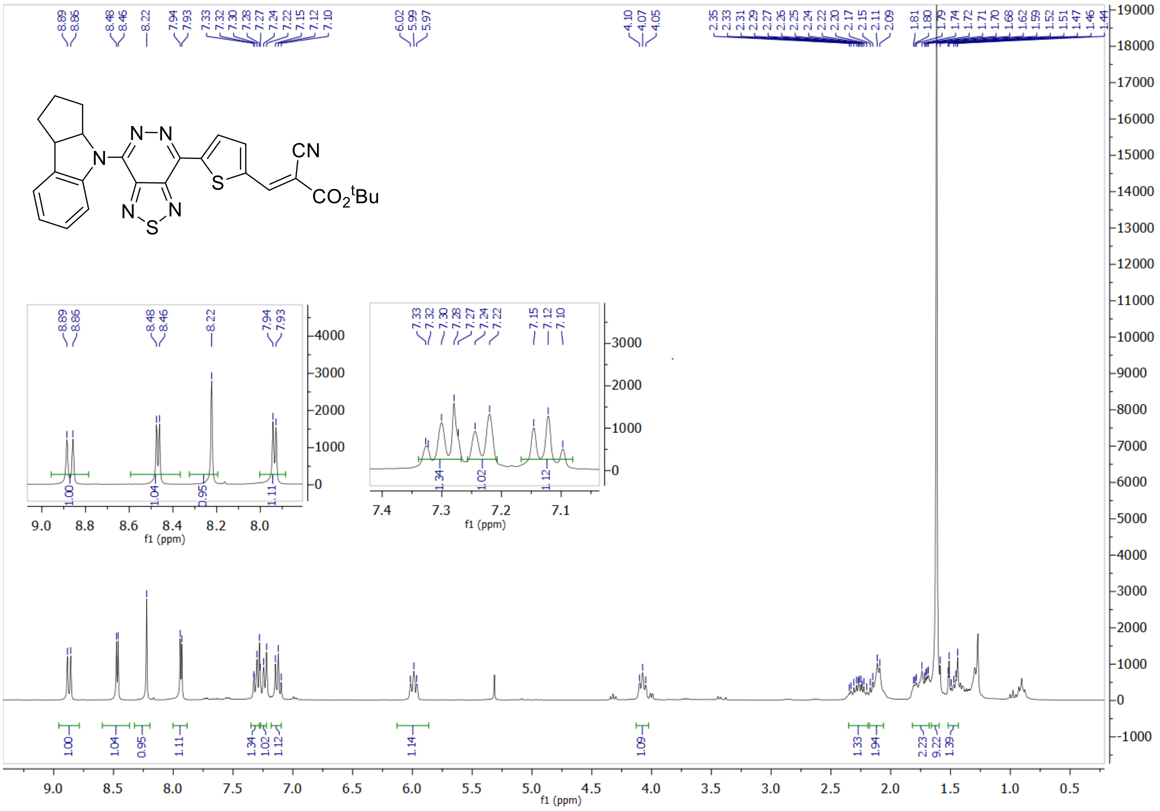

^{13}C NMR(75 MHz)

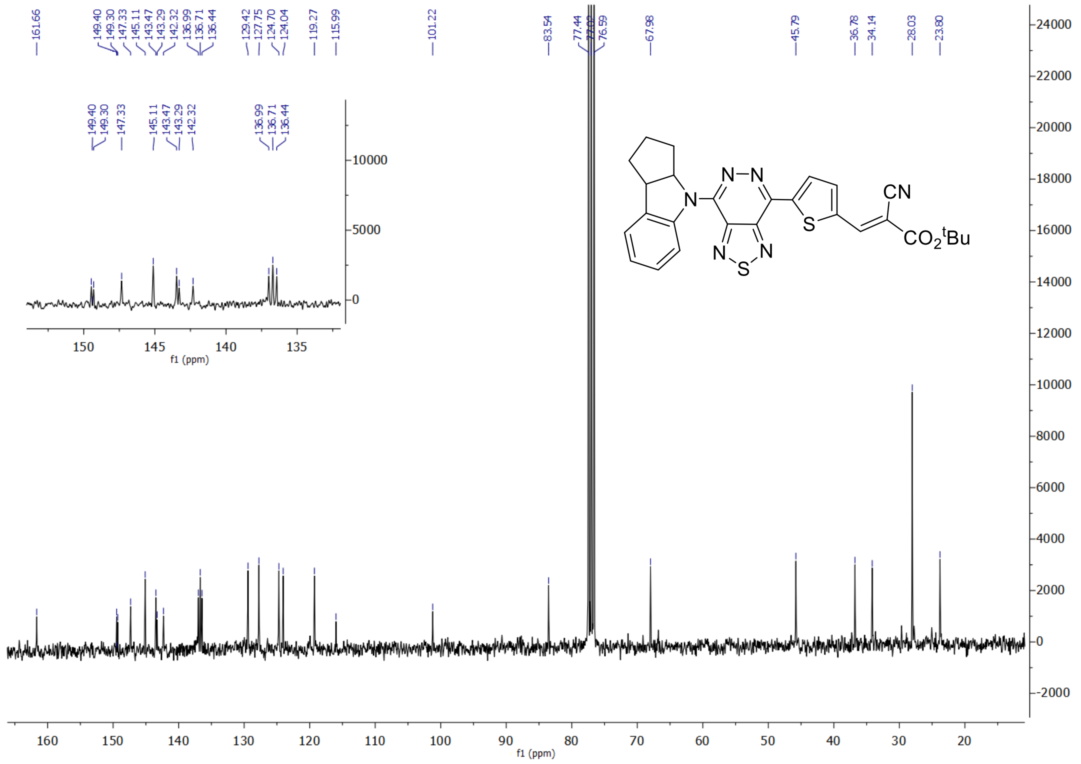

tert-Butyl 2-cyano-3-(5-(7-(2,3,4,4a-tetrahydro-1H-carbazol-9(9aH)-yl)-[1,2,5]thiadiazolo[3,4-d]pyridazin-4-yl)thiophen-2-yl)acrylate (7b)

^{1}H NMR(300 MHz)

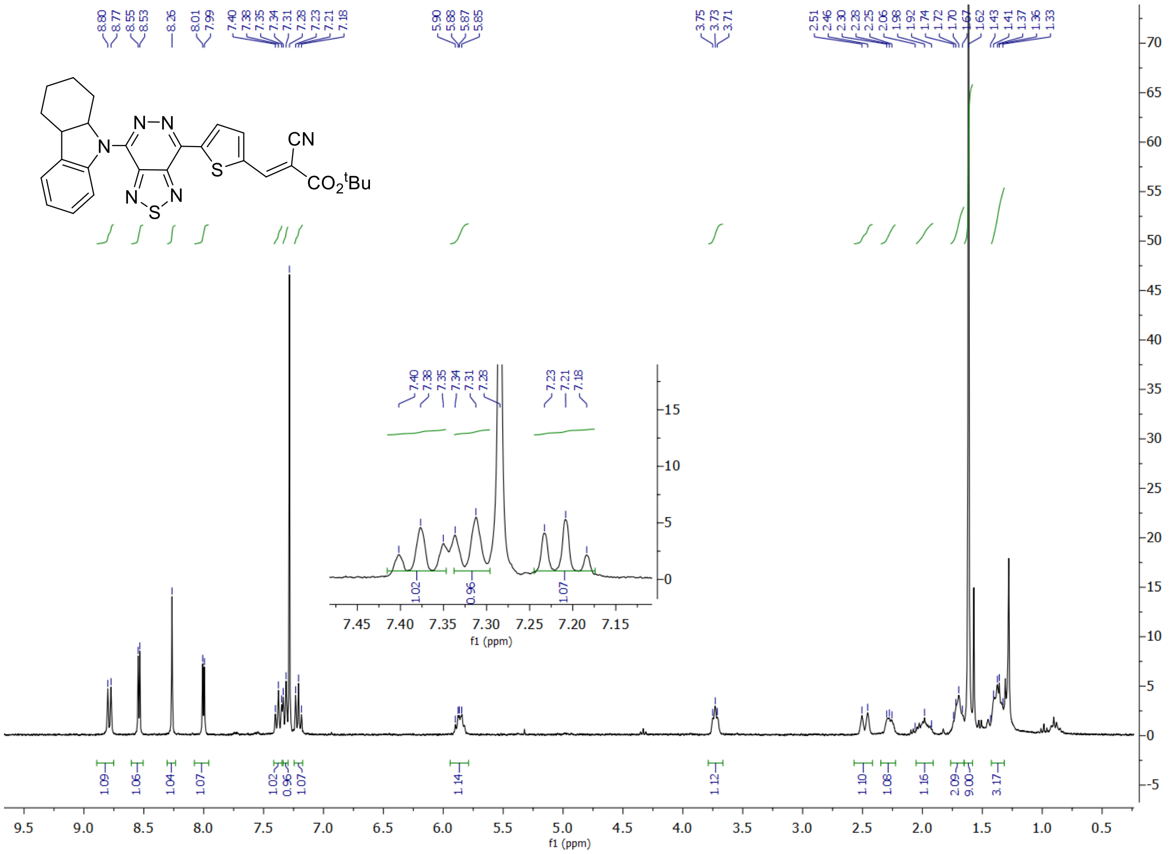

^{13}C NMR(75 MHz)

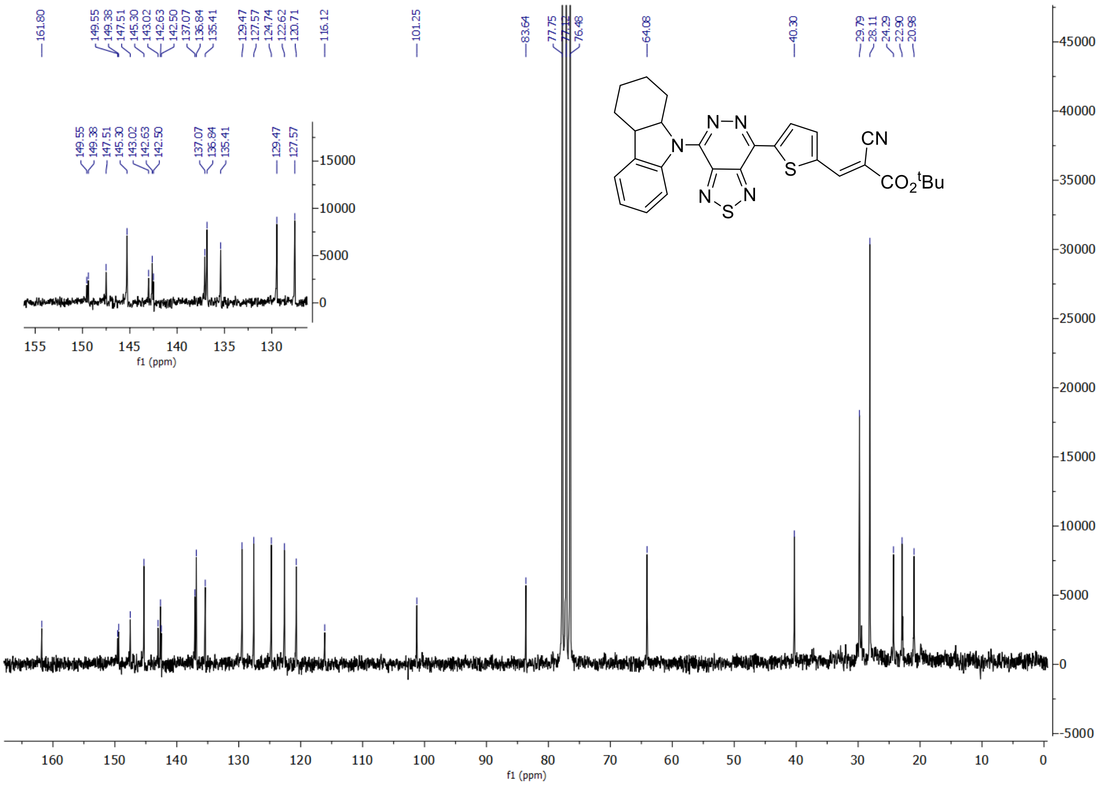

tert-Butyl 2-cyano-3-(5-(7-(1,2,3,4,4a,9a-hexahydro-9H-1,4-methanocarbazol-9-yl)-[1,2,5]thiadiazolo[3,4-d]pyridazin-4-yl)thiophen-2-yl)acrylate (7c)

^{1}H NMR(300 MHz)

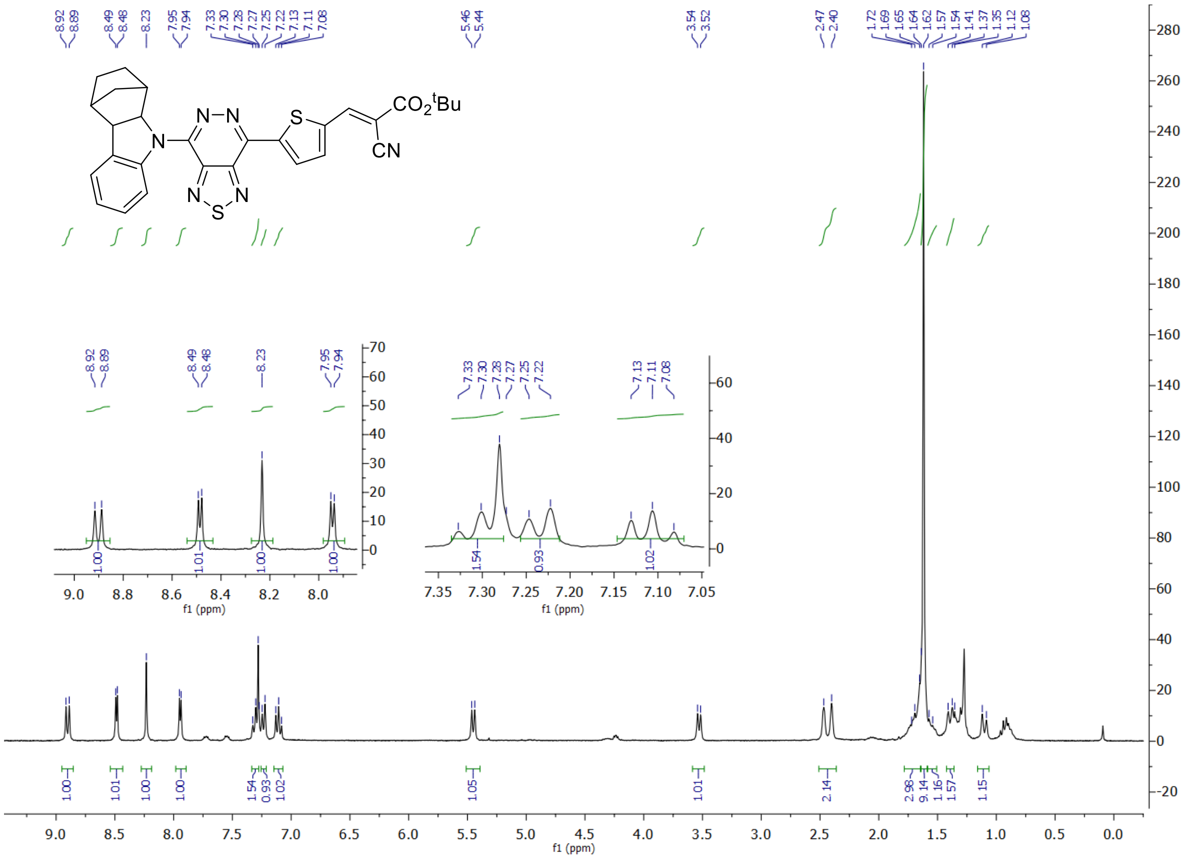

^{13}C NMR(75 MHz)

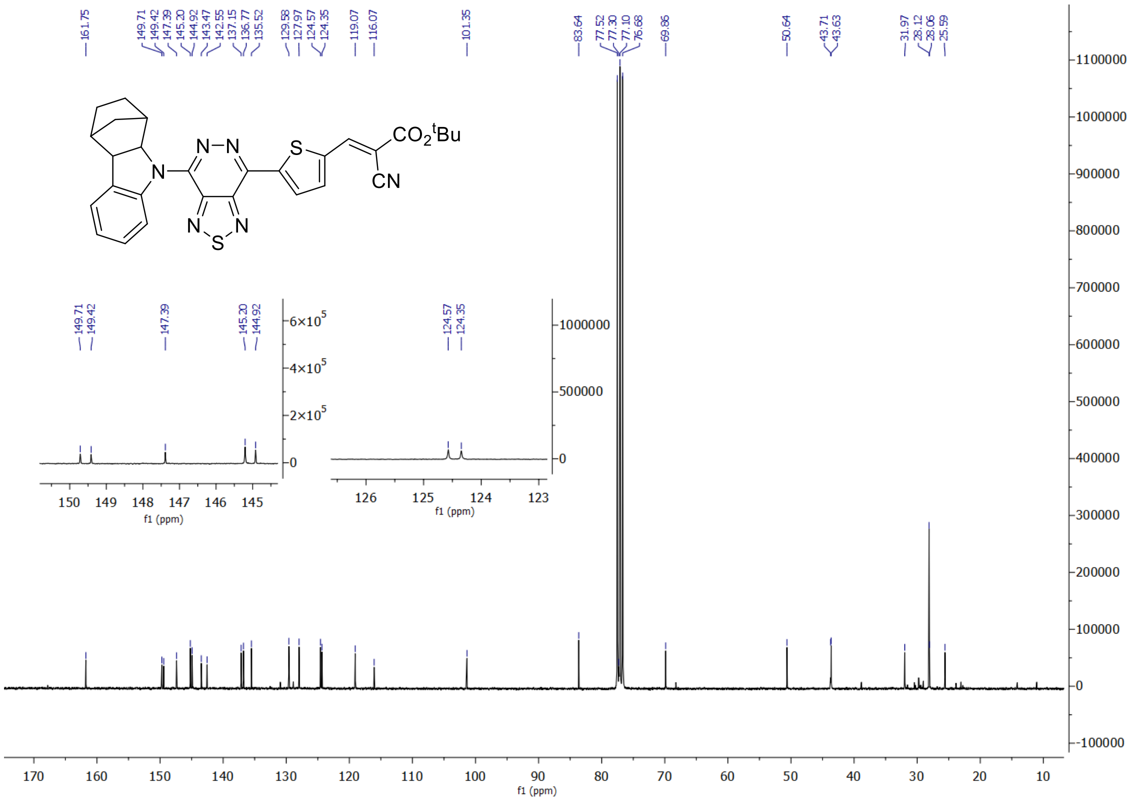

tert-Butil 2-cyano-3-(5-(7-(9-(p-tolyl)-2,3,4,4a,9,9a-hexahydro-1H-carbazol-6-yl)-[1,2,5]thiadiazolo[3,4-d]pyridazin-4-yl)thiophen-2-yl)acrylate (7d)

^{1}H NMR(300 MHz)

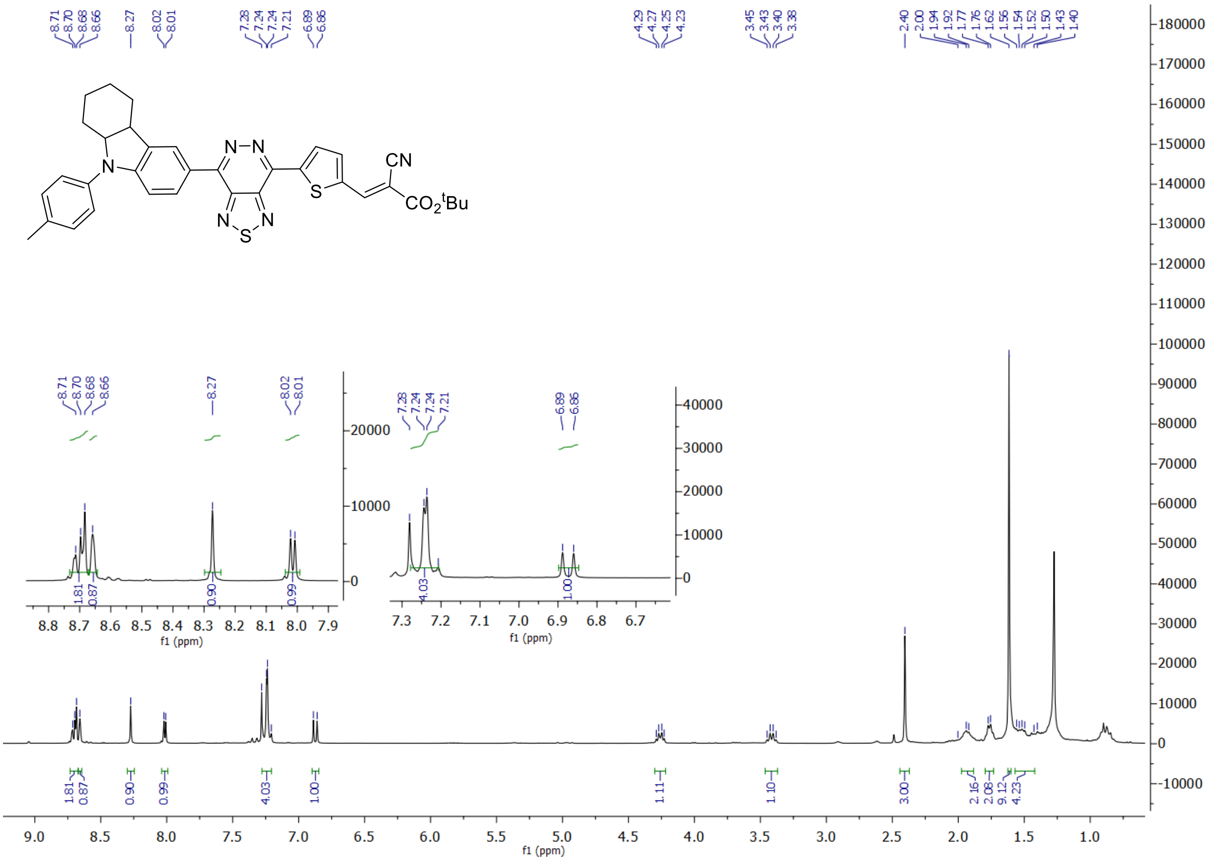

^{13}C NMR(75 MHz)

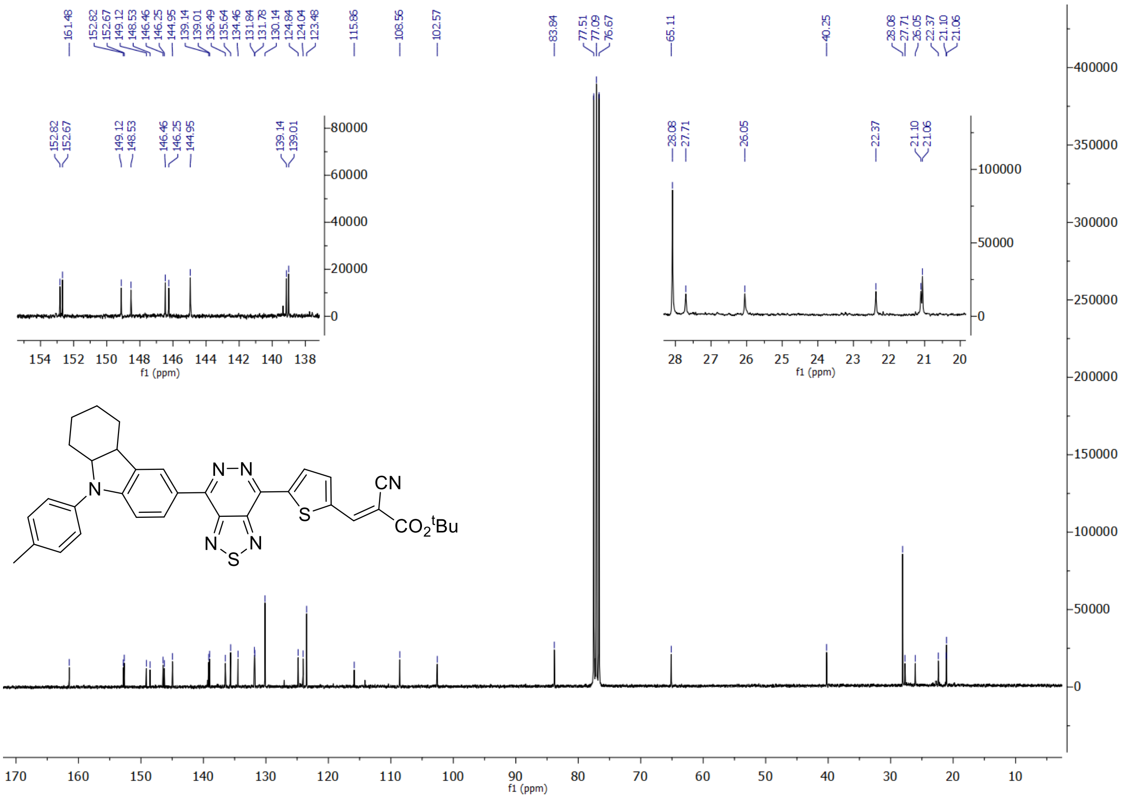

2-Cyano-3-(5-(7-(1,3,3a,8b-tetrahydrocyclopenta[b]indol-4(2H)-yl)-[1,2,5]thiadiazolo[3,4-d]pyridazin-4-yl)thiophen-2-yl)acrylic acid (TIM1)

^{1}H NMR(300 MHz)

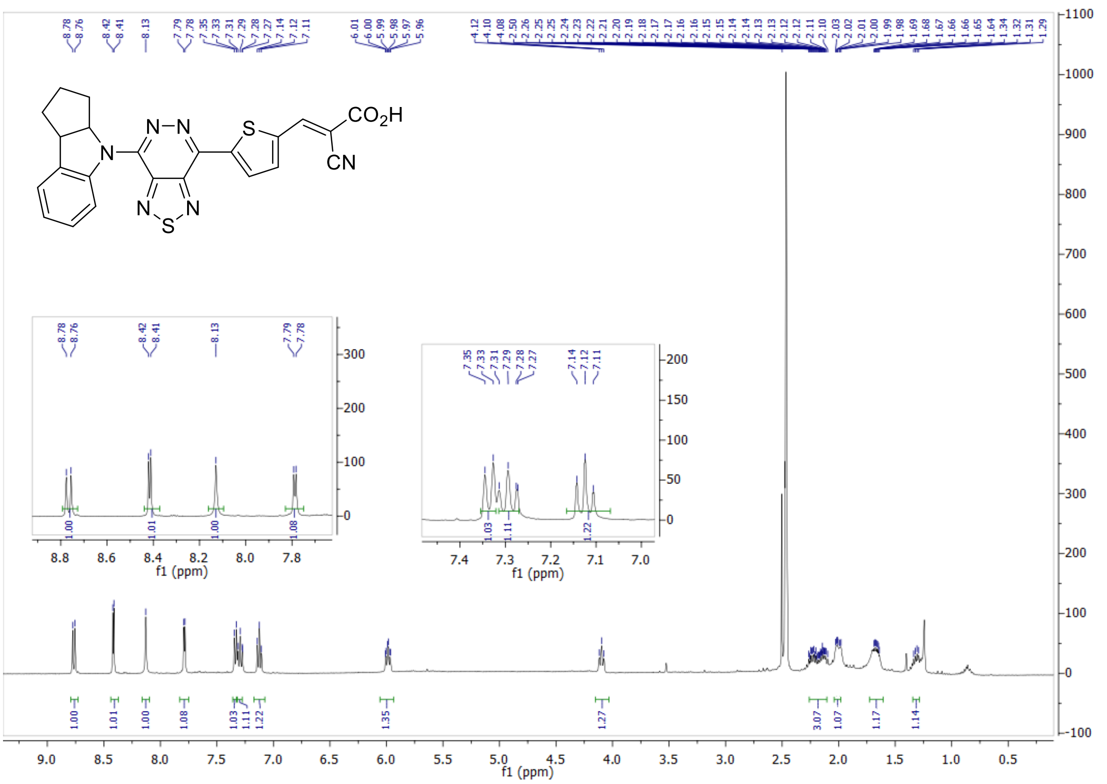

^{13}C NMR(75 MHz)

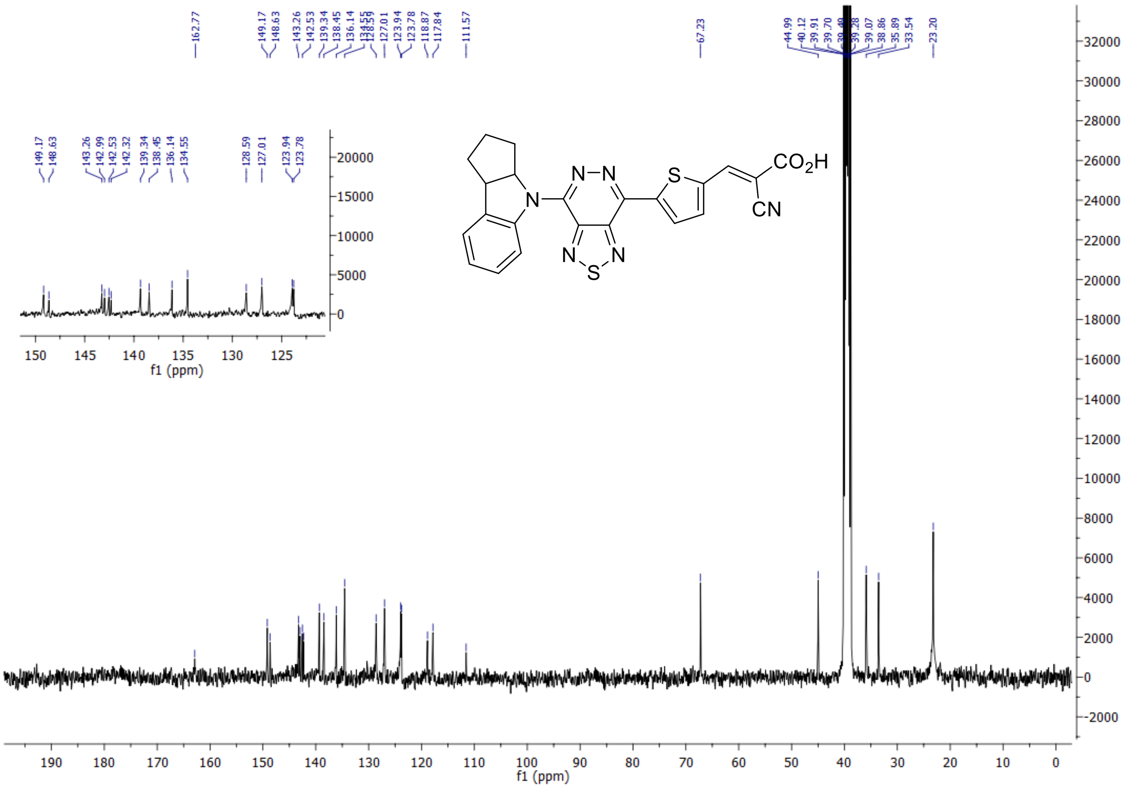

2-Cyano-3-(5-(7-(2,3,4,4a-tetrahydro-1H-carbazol-9(9aH)-yl)-[1,2,5]thiadiazolo[3,4-d]pyridazin-4-yl)thiophen-2-yl)acrylic acid (TIM2)

^{1}H NMR(300 MHz)

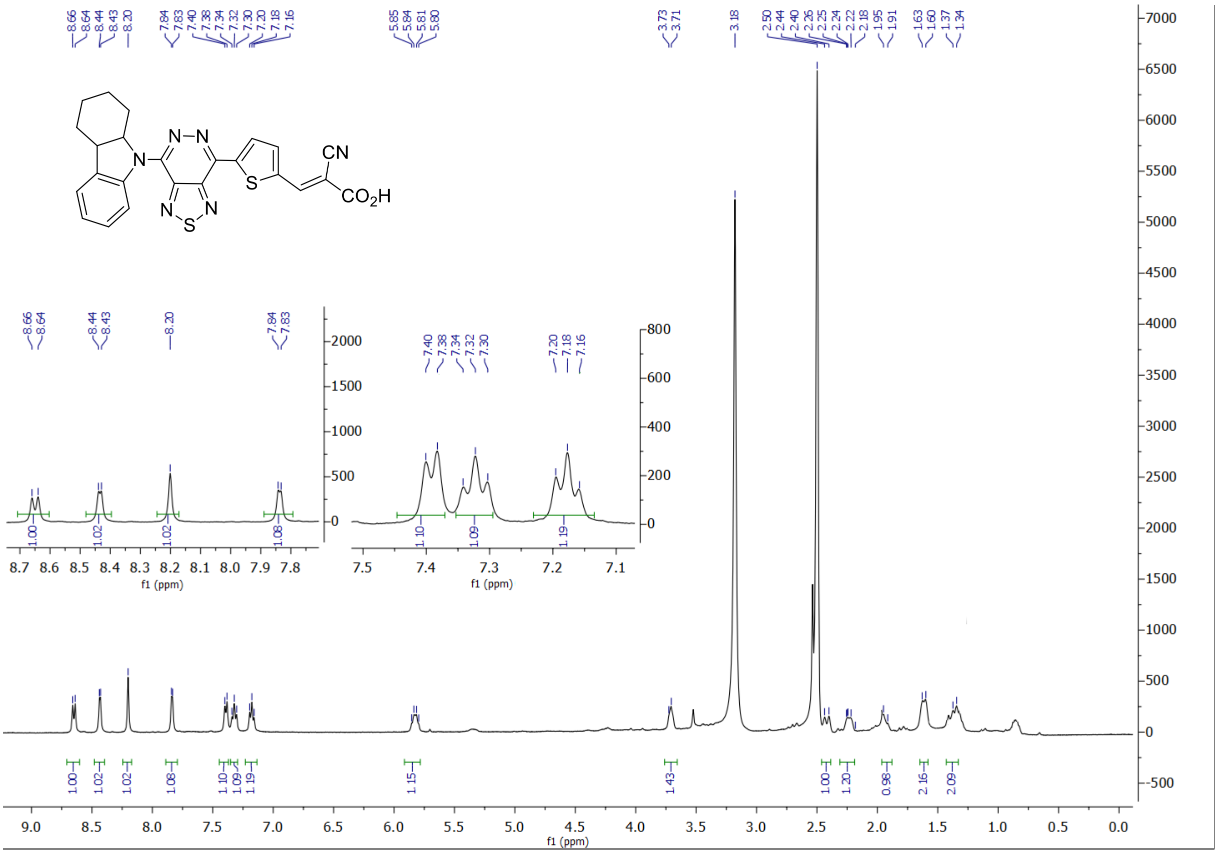

^{13}C NMR(75 MHz)

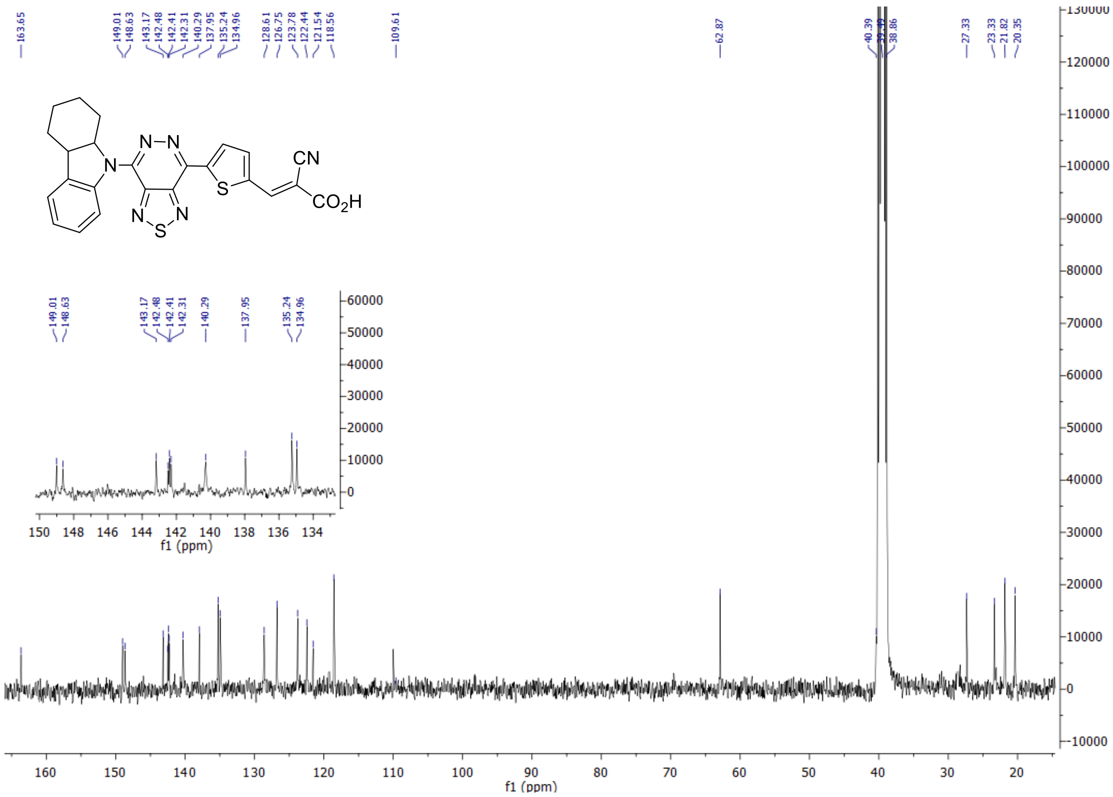

2-Cyano-3-(5-(7-(1,2,3,4,4a,9a-hexahydro-9H-1,4-methanocarbazol-9-yl)-[1,2,5]thiadiazolo[3,4-d]pyridazin-4-yl)thiophen-2-yl)acrylic acid (TIM3)

^{1}H NMR(300 MHz)

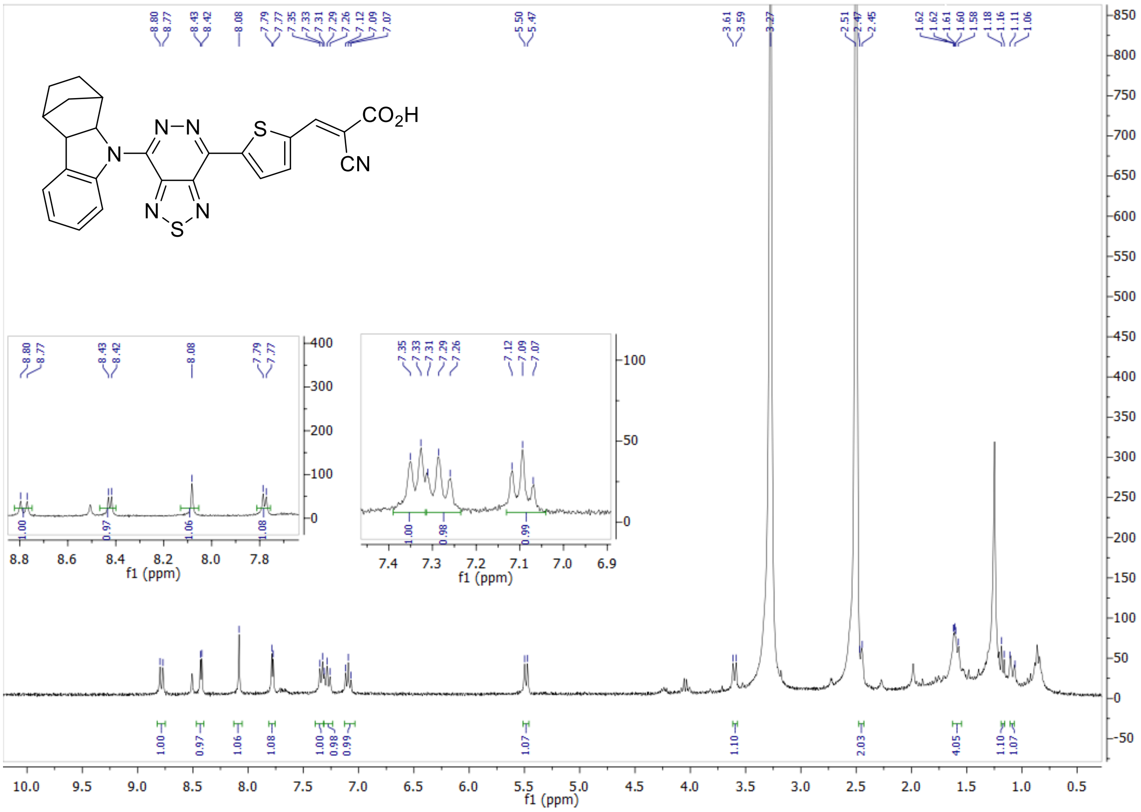

^{13}C NMR(75 MHz)

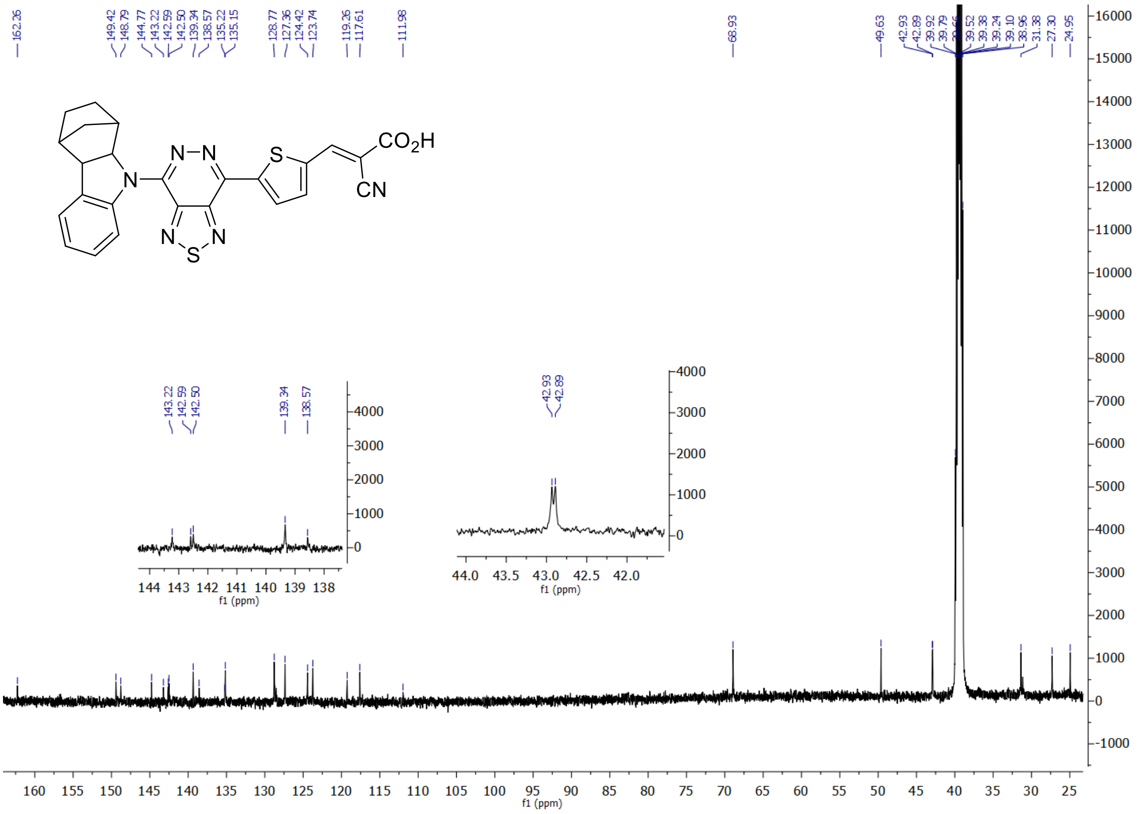

2-Cyano-3-(5-(7-(9-(p-tolyl)-2,3,4,4a,9,9a-hexahydro-1H-carbazol-6-yl)-[1,2,5]thiadiazolo[3,4-d]pyridazin-4-yl)thiophen-2-yl)acrylic acid (TIM4)

^{1}H NMR(300 MHz)

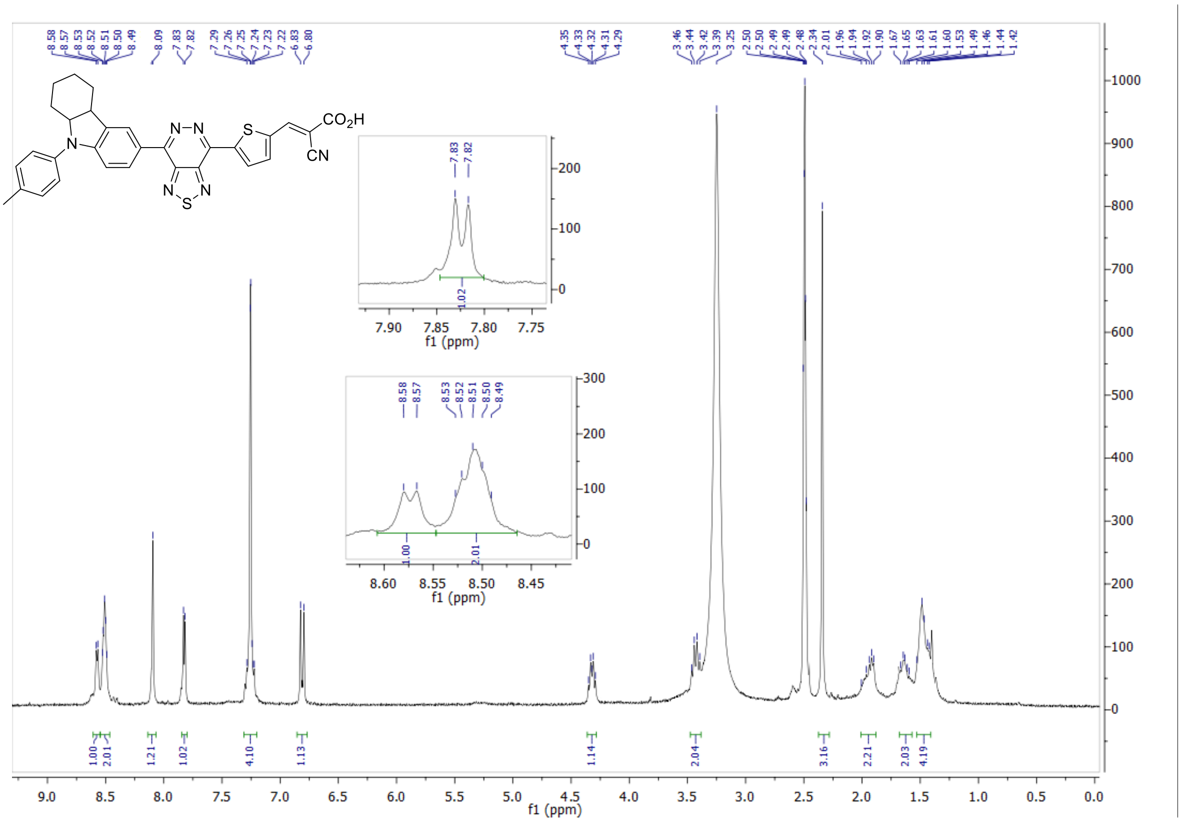

^{13}C NMR(75 MHz)

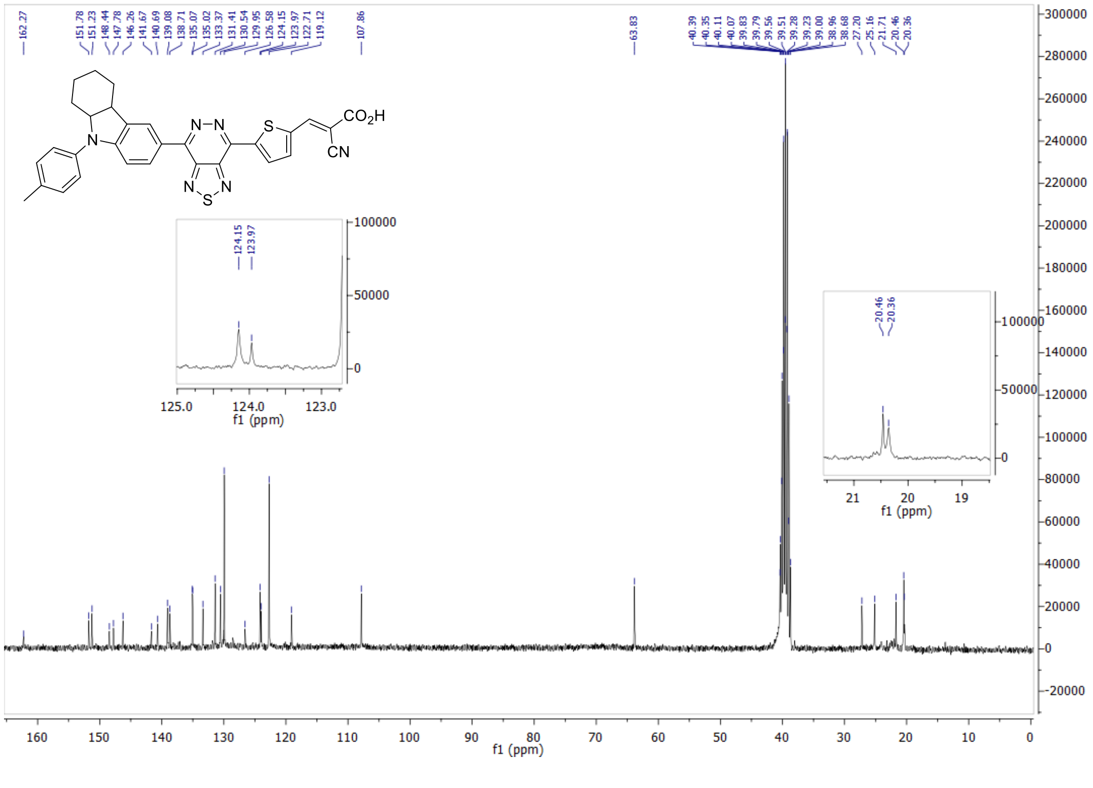

2. Quantum-chemical calculations

General information

Quantum-chemical calculations were performed with the Gaussian 16 Rev A.03 program.[1] DFT (or TD-DFT for excited states) wB97XD/6-31+g(d',p') level of theory was used for all calculations. All calculations were performed in gas phase. Cartesian coordinates are given in angstroms, absolute energies for all substances are given in hartrees. Analysis of vibrational frequencies was performed for all optimized structures. All compounds were characterized by only real vibrational frequencies. Wavefunction stability, using *stable* keyword,[2] was also checked for ground state of each molecule.

Ground state calculations

For calculations of optimized geometries, frequencies, dipole moments and MO orbitals, following keywords were used:

Optimization and frequencies:

# Opt freq wB97XD/6-31+g(d',p') nosymm

MO orbitals analysis:

%chk=1.chk
#P wB97XD/6-31+g(d',p') nosymm pop=(nboread,full) gfoldprint test 1

$NBO BNDIDX PLOT $END

Calculations of first excited state characteristics

Single point calculation was performed to obtain 20 lowest excited states of each compound. Keywords are:

# SP wB97XD/6-31+g(d',p') TD=NStates=20 nosymm

---

[1] Gaussian 16, Revision A.03, M. J. Frisch, G. W. Trucks, H. B. Schlegel, G. E. Scuseria, M. A. Robb, J. R. Cheeseman, G. Scalmani, V. Barone, G. A. Petersson, H. Nakatsuji, X. Li, M. Caricato, A. V. Marenich, J. Bloino, B. G. Janesko, R. Gomperts, B. Mennucci, H. P. Hratchian, J. V. Ortiz, A. F. Izmaylov, J. L. Sonnenberg, D. Williams-Young, F. Ding, F. Lipparini, F. Egidi, J. Goings, B. Peng, A. Petrone, T. Henderson, D. Ranasinghe, V. G. Zakrzewski, J. Gao, N. Rega, G. Zheng, W. Liang, M. Hada, M. Ehara, K. Toyota, R. Fukuda, J. Hasegawa, M. Ishida, T. Nakajima, Y. Honda, O. Kitao, H. Nakai, T. Vreven, K. Throssell, J. A. Montgomery, Jr., J. E. Peralta, F. Ogliaro, M. J. Bearpark, J. J. Heyd, E. N. Brothers, K. N. Kudin, V. N. Staroverov, T. A. Keith, R. Kobayashi, J. Normand, K. Raghavachari, A. P. Rendell, J. C. Burant, S. S. Iyengar, J. Tomasi, M. Cossi, J. M. Millam, M. Klene, C. Adamo, R. Cammi, J. W. Ochterski, R. L. Martin, K. Morokuma, O. Farkas, J. B. Foresman, and D. J. Fox, Gaussian, Inc., Wallingford CT, 2016.

[2] For detailed description of all keywords, basis sets and functionals please refer to Gaussian 16 manual or http://www.gaussian.com/keywords and references therein.

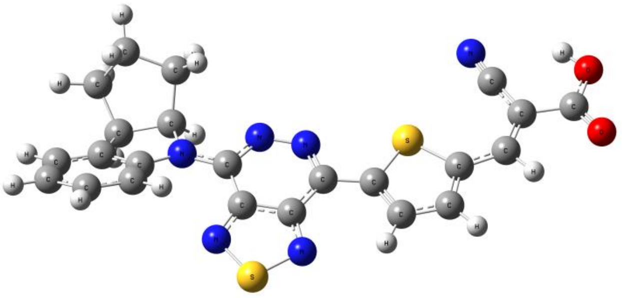

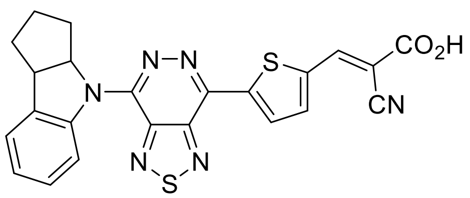

TIM1 ground state

C	-5.44993400	-0.32559000	-0.57785100
C	-4.90201100	-1.25162700	-1.54886900
C	-3.47678000	-1.36152900	-1.62664000
C	-2.68505800	-0.54596500	-0.74673400
N	-3.28724900	0.29836300	0.05582100
N	-4.61884700	0.41800600	0.14555200
N	-5.53707100	-1.97525400	-2.46484300
S	-4.39790000	-2.74809200	-3.32360200
N	-3.06113600	-2.18808900	-2.58344000
N	-6.77269800	-0.06294200	-0.41602700
C	-1.22502000	-0.57191000	-0.70332700
C	-0.35087900	-1.35651700	-1.42898200
C	0.99422200	-1.09437100	-1.10308800
C	1.14296600	-0.11548900	-0.13493900
S	-0.40325100	0.49268900	0.38302800
C	2.42271100	0.31290500	0.34856200
C	2.75519800	1.24464000	1.27825700
C	-7.18982300	1.21863000	0.19262800
C	-8.67847500	1.37578300	-0.22610100
C	-9.05243000	-0.02956900	-0.63501800
C	-7.91647100	-0.84184500	-0.70483700
C	-7.17884400	1.22744000	1.74230000
C	-8.65143400	1.13949300	2.17250100
C	-9.39904700	1.85781300	1.04387400
C	-10.31069700	-0.56827900	-0.85722500
C	-10.43375700	-1.93369400	-1.12437800
C	-9.29913200	-2.74393100	-1.13744600
C	-8.02760000	-2.21295000	-0.91820700
C	4.21416400	1.46658700	1.56737600
O	5.08387800	0.85354000	1.00767700
O	4.51292200	2.39196100	2.49055000
C	1.80817700	2.03007900	2.00197800
N	1.11867800	2.71425000	2.63912000
H	-0.67886100	-2.08252500	-2.16203000
H	1.84095300	-1.59977900	-1.55664700
H	3.27786100	-0.19055400	-0.10297900
H	-6.53894600	1.99890100	-0.20638900
H	-8.80333200	2.07156600	-1.06439600
H	-6.73633900	2.17098400	2.08075200
H	-6.55439500	0.42460300	2.14113100
H	-8.97474500	0.09195500	2.22225500
H	-8.83101300	1.58729500	3.15480400
H	-9.28065900	2.94476900	1.14273700
H	-10.47279600	1.64289000	1.02364700
H	-11.19359600	0.06480100	-0.80628000
H	-11.41385700	-2.36827700	-1.29886700
H	-9.40050000	-3.81157200	-1.31232200
H	-7.16157600	-2.86184400	-0.91823300

H 3.71946000 2.80224300 2.85514700

DFT wB97XD/6-31+g(d',p'), gas phase		
Sum of electronic and zero-point Energies=	-2160.565596	$E_0 + E_{ZPE}$
Sum of electronic and thermal Energies=	-2160.539040	$E_0 + E_{tot}$
Sum of electronic and thermal Enthalpies=	-2160.538096	$E_0 + H_{corr}$
Sum of electronic and thermal Free Energies=	-2160.625873	$E_0 + G_{corr}$
Zero-point correction *(unscaled)* =	0.362192	

MO (number)	Energy, eV	Representation
LUMO+1 (124)	-1.121	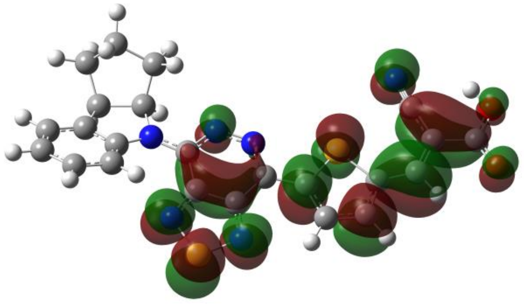
LUMO (123)	-1.924	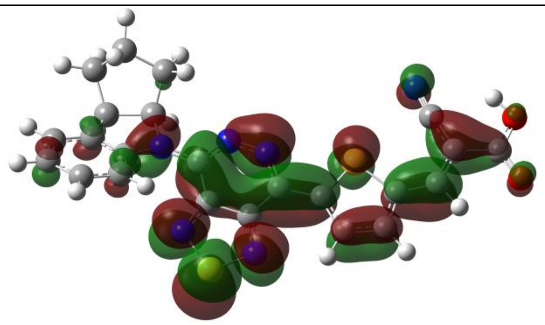
HOMO (122)	-7.640	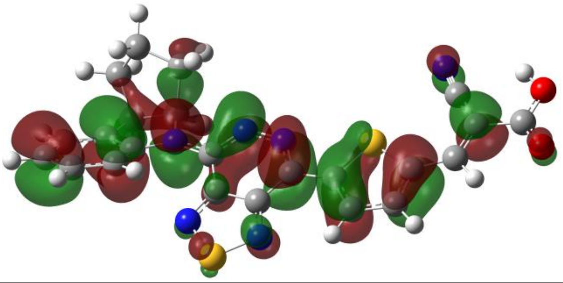
HOMO-1 (121)	-8.697	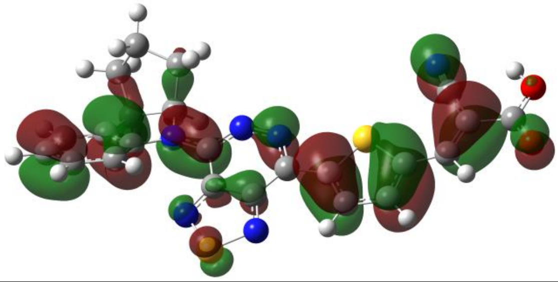

Calculated characteristics of the **first excited state** in gas phase. Vertical excitation, single point TD-DFT wB97XD/6-31+g(d',p') level of theory.

λ_{abs} vertical nm	f	Orbitals (contribution)	E_{gap} eV	μ S_0 D	μ S_1 vertical D	$\Delta\mu(S_{1\ vert}-S_0)$ D
474	0.6044	HOMO-LUMO (91%)<br>HOMO-LUMO+1 (3%)	5.72	9.89	9.89	0

		HOMO-1-LUMO+1 (2%)				

RAW DATA for five lowest excited states

Excitation energies and oscillator strengths:

Excited State 1: Singlet-?Sym 2.6159 eV 473.97 nm f=0.6044 <S**2>=0.000

121 ->124 -0.10402

122 ->123 0.67284

122 ->124 -0.12847

Total Energy, E(TD-HF/TD-DFT) = -2160.83165753

Excited State 2: Singlet-?Sym 3.2115 eV 386.06 nm f=0.0120 <S**2>=0.000

119 ->123 0.55054

120 ->123 -0.37656

Excited State 3: Singlet-?Sym 3.5079 eV 353.44 nm f=0.5531 <S**2>=0.000

121 ->123 -0.37961

121 ->124 -0.20436

122 ->124 0.52020

Excited State 4: Singlet-?Sym 4.0203 eV 308.40 nm f=0.0542 <S**2>=0.000

115 ->123 -0.10104

117 ->123 -0.10998

117 ->124 0.12054

121 ->123 0.52082

121 ->124 -0.23113

122 ->124 0.30705

Excited State 5: Singlet-?Sym 4.2414 eV 292.32 nm f=0.0011 <S**2>=0.000

118 ->123 0.54781

118 ->124 0.32862

121 ->124 -0.14613

122 ->124 -0.11658

122 ->125 0.15625

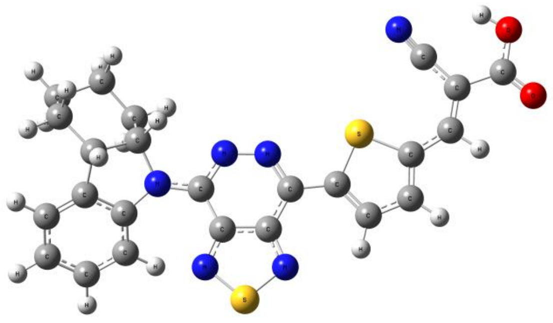

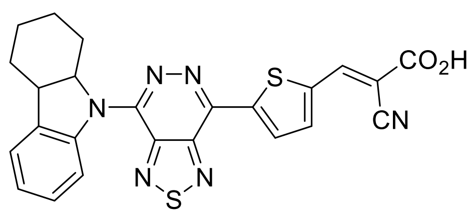

TIM2 ground state

C	-5.41552100	-0.80897600	-0.09506000
C	-4.87834700	-2.15365600	-0.14625900
C	-3.47078300	-2.31226400	0.05407800
C	-2.68549700	-1.13268600	0.29697900
N	-3.28545200	0.03138900	0.36887500
N	-4.60156000	0.20321000	0.18333300
N	-5.52783200	-3.30533700	-0.27789600
S	-4.41433400	-4.48178500	-0.17989700
N	-3.07601300	-3.58351800	0.04583500
N	-6.73471600	-0.51293800	-0.20483500
C	-1.23847800	-1.14356900	0.49754500
C	-0.36962200	-2.21637900	0.47382700
C	0.96335900	-1.82579400	0.70795100
C	1.10821100	-0.46324900	0.90876400
S	-0.42672200	0.35087200	0.80729100
C	2.37543000	0.15571700	1.16510200
C	2.70150800	1.45544700	1.38311500
C	-7.27168800	0.78356200	0.27555900
C	-8.73550200	0.40455500	0.60025900
C	-9.00354400	-0.63741300	-0.46344300
C	-7.78340800	-1.18558200	-0.87994200
C	-10.19134800	-1.07089300	-1.02734200
C	-10.15472300	-2.05039500	-2.02540000
C	-8.93317000	-2.56558900	-2.45365000
C	-7.72791100	-2.13286100	-1.89480800
C	-7.14617600	1.86041600	-0.81282600
C	-8.06983400	3.05085500	-0.55252900
C	-9.52562800	2.58987200	-0.46148100
C	-9.69572200	1.59320400	0.68597800
C	4.14693100	1.79121900	1.62646100
O	5.01141200	0.95567900	1.63461800
O	4.44007000	3.08246900	1.83798000
C	1.75912600	2.52747600	1.39611500
N	1.07215100	3.46376000	1.42613200
H	-0.69320400	-3.23425500	0.29683500
H	1.80429100	-2.51161900	0.73207800
H	3.22500600	-0.52705500	1.19086400
H	-6.71514000	1.08490700	1.16702200
H	-8.73780100	-0.11340500	1.57167200
H	-11.14011100	-0.64585700	-0.70845500
H	-11.07843100	-2.39665800	-2.48030400
H	-8.90928100	-3.30789000	-3.24681800
H	-6.78687800	-2.52900000	-2.25558000

H	-6.09799500	2.16743300	-0.87491200
H	-7.41456300	1.41215700	-1.78003800
H	-7.94763400	3.78956100	-1.35285500
H	-7.78327400	3.55219200	0.38407000
H	-10.19447100	3.44489200	-0.30818700
H	-9.81829700	2.12032200	-1.41157800
H	-10.72787000	1.22411900	0.73699600
H	-9.51128900	2.11478500	1.63548600
H	3.65245300	3.63855200	1.80327000

DFT wB97XD/6-31+g(d',p'), gas phase		
Sum of electronic and zero-point Energies=	-2199.847637	$E_0 + E_{ZPE}$
Sum of electronic and thermal Energies=	-2199.820083	$E_0 + E_{tot}$
Sum of electronic and thermal Enthalpies=	-2199.819139	$E_0 + H_{corr}$
Sum of electronic and thermal Free Energies=	-2199.908898	$E_0 + G_{corr}$
Zero-point correction *(unscaled)* =	0.391694	

MO (number)	Energy, eV	Representation
LUMO+1 (128)	-1.115	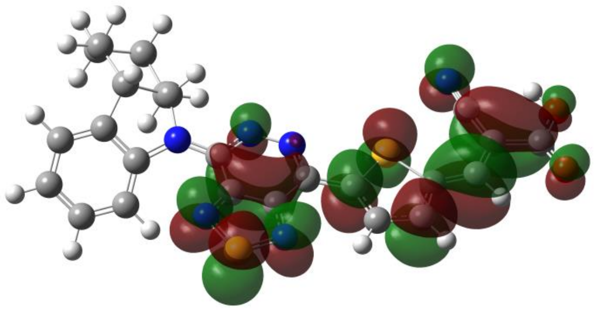
LUMO (127)	-1.914	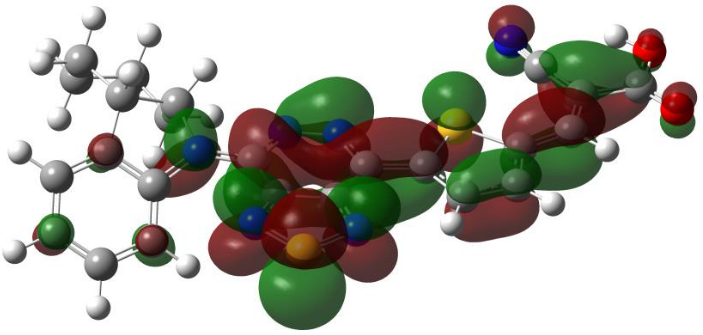
HOMO (126)	-7.639	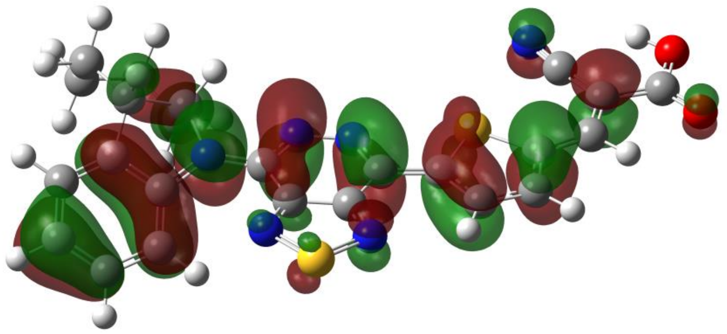
HOMO-1 (125)	-8.682	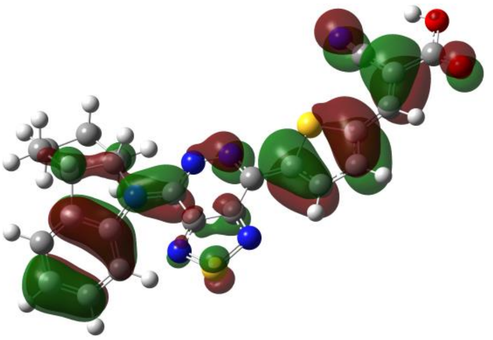

Calculated characteristics of the **first excited state** in gas phase. Vertical excitation, single point TD-DFT wB97XD/6-31+g(d',p') level of theory.

λ_{abs} vertical nm	f	Orbitals (contribution)	E_{gap} eV	μ S_0 D	μ S_1 vertical D	Δμ($S_{1\ vert}$-S_0) D
474	0.6011	HOMO-LUMO (90%)<br>HOMO-LUMO+1 (3%)<br>HOMO-1-LUMO+1 (2%)	5.72	9.93	9.93	0

RAW DATA for five lowest excited states

```
Excitation energies and oscillator strengths:

 Excited State   1:      Singlet-?Sym    2.6167 eV  473.81 nm  f=0.6011  <S**2>=0.000

     125 ->128         0.10248

     126 ->127         0.67234

     126 ->128         0.12861

Total Energy, E(TD-HF/TD-DFT) =  -2200.14316768

Excited State   2:      Singlet-?Sym    3.2322 eV  383.59 nm  f=0.0141  <S**2>=0.000

     123 ->127         0.66394

     123 ->129        -0.11469

     125 ->127         0.10380

 Excited State   3:      Singlet-?Sym    3.5085 eV  353.38 nm  f=0.5543  <S**2>=0.000

     125 ->127         0.37256

     125 ->128        -0.20505

     126 ->128         0.52220

Excited State   4:      Singlet-?Sym    4.0196 eV  308.45 nm  f=0.0546  <S**2>=0.000

     121 ->127         0.11151

     121 ->128         0.11803

     125 ->127         0.52231

     125 ->128         0.23462

     126 ->128        -0.30055

 Excited State   5:      Singlet-?Sym    4.2441 eV  292.13 nm  f=0.0010  <S**2>=0.000

     122 ->127         0.54942

     122 ->128        -0.33076

     125 ->128        -0.14036
```

126 ->128 -0.11832

126 ->129 -0.15236

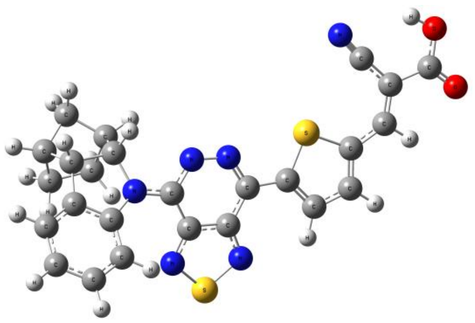

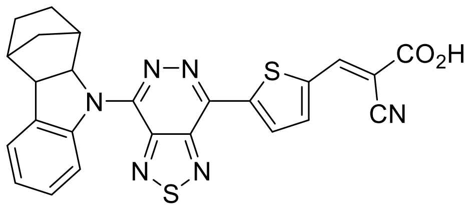

TIM3 ground state

C	-5.60895000	-0.68720600	-0.03222100
C	-4.96013300	-1.62329300	-0.92641400
C	-3.53158500	-1.69201700	-0.89346900
C	-2.83582600	-0.83035600	0.02240800
N	-3.52260400	0.02374800	0.74331400
N	-4.85962300	0.11304400	0.71516500
N	-5.50261100	-2.37676000	-1.87818900
S	-4.27912000	-3.12947900	-2.63182500
N	-3.01979400	-2.52178000	-1.80021700
N	-6.95094100	-0.45778800	0.00759500
C	-1.38467100	-0.81777200	0.18829700
C	-0.43715100	-1.61728700	-0.41933100
C	0.86975900	-1.30945500	0.00676800
C	0.91624600	-0.27997700	0.93181900
S	-0.67933400	0.31816400	1.28404300
C	2.14167600	0.20212800	1.49927300
C	2.37523900	1.19068400	2.39956800
C	-7.43449600	0.87949800	0.40907600
C	-8.98130000	0.80945500	0.24441100
C	-9.24302900	-0.62827100	-0.09673800
C	-8.03873300	-1.33578200	-0.20160800
C	-10.45686800	-1.28273300	-0.24055200
C	-10.47216900	-2.66015300	-0.47027300
C	-9.27024200	-3.36366100	-0.52574500
C	-8.04167600	-2.71675100	-0.38524000
C	-7.01728400	2.03547600	-0.53105700
C	-7.16612300	1.59890400	-2.00093900
C	-8.70895200	1.48272200	-2.18643100
C	-9.26319000	1.90633600	-0.81276500
C	-8.23568500	2.96686200	-0.37167200
C	3.79970000	1.45976400	2.80044000
O	4.72443200	0.83412500	2.35458400
O	4.00185400	2.44214600	3.69007700
C	1.35529100	1.99500200	2.99149500
N	0.60086900	2.69937700	3.52447100
H	-0.68692100	-2.38565400	-1.13998100
H	1.76153300	-1.81800200	-0.34563300
H	3.04189600	-0.30607200	1.15278700
H	-7.11991900	1.08563800	1.43591400
H	-9.48795800	1.07690600	1.17892000
H	-11.38703900	-0.72507900	-0.15920400
H	-11.41641900	-3.18485800	-0.58357600
H	-9.28151500	-4.44006800	-0.67439800
H	-7.12538600	-3.29006300	-0.42424900
H	-6.04308700	2.46003300	-0.28126400

H	-6.74384600	2.36834300	-2.65606100
H	-6.64693800	0.66204500	-2.22511900
H	-9.06724700	2.16567200	-2.96414500
H	-9.02388100	0.47245000	-2.46711400
H	-10.30996000	2.22259700	-0.83347600
H	-8.38648200	3.31727700	0.65642800
H	-8.19270000	3.83556400	-1.03841400
H	3.17351500	2.85633300	3.96045400

DFT wB97XD/6-31+g(d',p'), gas phase		
Sum of electronic and zero-point Energies=	-2237.925809	$E_0 + E_{ZPE}$
Sum of electronic and thermal Energies=	-2237.898429	$E_0 + E_{tot}$
Sum of electronic and thermal Enthalpies=	-2237.897485	$E_0 + H_{corr}$
Sum of electronic and thermal Free Energies=	-2237.987018	$E_0 + G_{corr}$
Zero-point correction *(unscaled)* =	0.398728	

MO (number)	Energy, eV	Representation
LUMO+1 (131)	-1.149	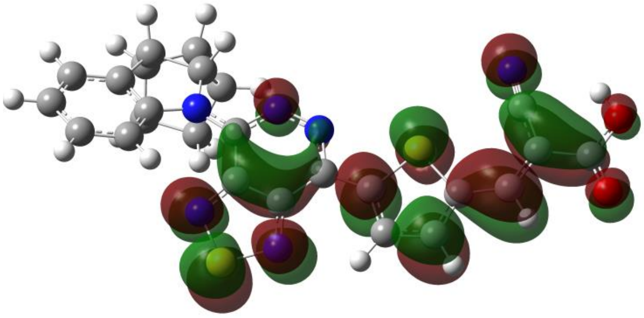
LUMO (130)	-1.979	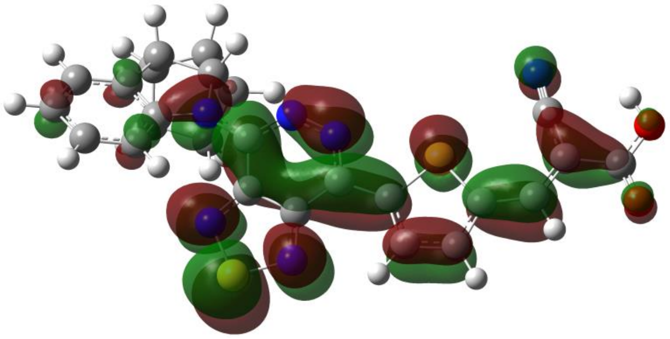
HOMO (129)	-7.609	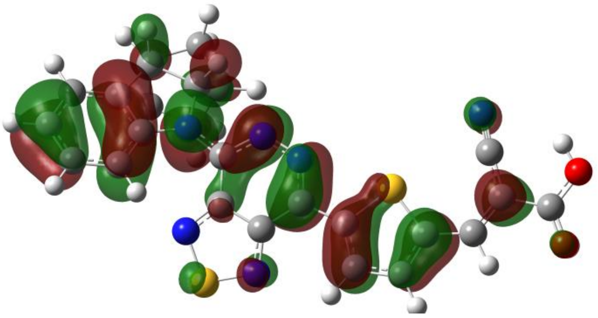

Calculated characteristics of the **first excited state** in gas phase. Vertical excitation, single point TD-DFT wB97XD/6-31+g(d',p') level of theory.

λ_{abs} vertical nm	f	Orbitals (contribution)	E_{gap} eV	μ S_0 D	μ S_1 vertical D	Δμ($S_{1\ vert}$-S_0) D
489	0.5332	HOMO-LUMO (89%)<br>HOMO-LUMO+1 (4%)	5.63	9.60	9.60	0

RAW DATA for five lowest excited states

Excitation energies and oscillator strengths:

Excited State 1: Singlet-?Sym 2.5370 eV 488.71 nm f=0.5332 <S**2>=0.000

129 ->130 0.66824

129 ->131 0.14098

Total Energy, E(TD-HF/TD-DFT) = -2238.23130451

Excited State 2: Singlet-?Sym 3.1909 eV 388.55 nm f=0.0502 <S**2>=0.000

126 ->130 0.65305

126 ->132 0.10183

126 ->133 0.10040

128 ->130 -0.15083

Excited State 3: Singlet-?Sym 3.4847 eV 355.80 nm f=0.5569 <S**2>=0.000

126 ->130 0.12205

128 ->130 0.40413

128 ->131 -0.21030

129 ->131 0.48436

129 ->132 -0.10279

Excited State 4: Singlet-?Sym 3.9595 eV 313.13 nm f=0.0534 <S**2>=0.000

124 ->130 -0.11004

124 ->131 -0.11738

128 ->130 0.49378

128 ->131 0.24704

129 ->131 -0.33465

129 ->133 0.10486

Excited State 5: Singlet-?Sym 4.2229 eV 293.60 nm f=0.0020 <S**2>=0.000

125 ->130 0.53322

125 ->131 -0.31462

128 ->131 -0.16536

129 ->131 -0.12291

129 ->132 0.18729

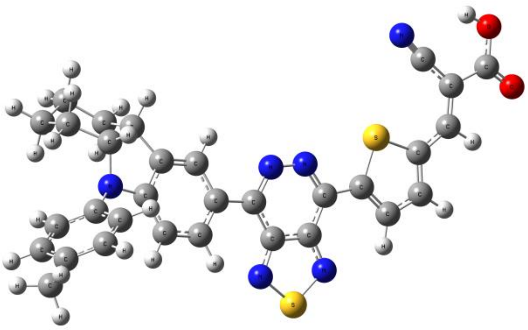

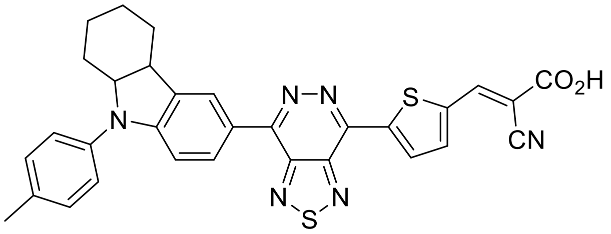

TIM4 ground state

C	-0.66209300	-0.83791300	-0.31502300
C	0.09569000	-1.95584600	-0.83492300
C	1.52083600	-1.92164600	-0.72243900
C	2.11481600	-0.76755600	-0.10865800
N	1.34159200	0.20382200	0.31580300
N	0.00001700	0.16950900	0.22580700
N	-0.35022500	-3.05510100	-1.44504500
S	0.95108300	-3.94731500	-1.81759200
N	2.12836400	-2.99031600	-1.23590800
C	-2.12638200	-0.72596800	-0.35881800
C	-2.71580900	0.53626300	-0.11231300
C	-4.08449500	0.66060800	-0.11606800
C	-4.90654600	-0.45485600	-0.36744900
C	-4.34940600	-1.70735600	-0.61866500
C	-2.96120000	-1.82608300	-0.61343800
C	-4.97443000	1.87530300	-0.00145400
C	-6.32280600	1.21430000	0.36849500
N	-6.24338500	-0.08519400	-0.34466000
C	-7.26634200	-1.05331100	-0.12102900
C	3.55337600	-0.58790600	0.07235400
C	4.58371000	-1.43902500	-0.27427000
C	5.84483000	-0.91067000	0.06387300
C	5.77259100	0.33556400	0.66378600
S	4.12322900	0.86535700	0.81501900
C	6.93461000	1.06178500	1.08939700
C	7.05451200	2.27674100	1.68143600
C	-7.25529100	-1.86624100	1.01601700
C	-8.27796100	-2.78485600	1.22786900
C	-9.33647900	-2.91129400	0.32014700
C	-9.33865000	-2.08787900	-0.80871800
C	-8.31322600	-1.17073800	-1.03211300
C	-10.42621500	-3.92925900	0.54603400
C	-5.02093300	2.62065400	-1.35280800
C	-6.24927200	3.51908700	-1.49415400
C	-7.52631700	2.69241200	-1.33197200
C	-7.56603500	2.03698800	0.04824700
C	8.43750000	2.77053500	2.00924800
O	9.42524600	2.13038600	1.76506700
O	8.52663100	3.97321600	2.59441100

C	5.95225000	3.12062200	2.01450600
N	5.12584500	3.87631900	2.32297700
H	-2.07437300	1.39109500	0.08020800
H	-4.97715200	-2.57378200	-0.80461400
H	-2.52531800	-2.80018800	-0.80182300
H	-4.66407400	2.56499000	0.79176700
H	-6.31691000	1.00036300	1.45292000
H	4.42241000	-2.39745800	-0.75122000
H	6.78731100	-1.41625000	-0.12078100
H	7.88460700	0.55820500	0.90901900
H	-6.43921500	-1.77729900	1.72975000
H	-8.25708600	-3.41233200	2.11684500
H	-10.15149000	-2.16551100	-1.52783300
H	-8.31474800	-0.53712000	-1.91511000
H	-10.68859300	-4.00179800	1.60681300
H	-11.33247700	-3.67466600	-0.01233800
H	-10.10207200	-4.92466100	0.21809100
H	-4.09278600	3.19297800	-1.46864200
H	-5.03435400	1.87832300	-2.16281400
H	-6.23396100	4.01706800	-2.47089200
H	-6.22689100	4.31313200	-0.73242900
H	-8.41585700	3.31890000	-1.46724300
H	-7.55635600	1.91894900	-2.11248100
H	-8.45866800	1.41050200	0.16622300
H	-7.63825500	2.82261300	0.81352100
H	7.65809100	4.37080600	2.73010100

DFT wB97XD/6-31+g(d',p'), gas phase		
Sum of electronic and zero-point Energies=	-2470.032296	$E_0 + E_{ZPE}$
Sum of electronic and thermal Energies=	-2469.997932	$E_0 + E_{tot}$
Sum of electronic and thermal Enthalpies=	-2469.996988	$E_0 + H_{corr}$
Sum of electronic and thermal Free Energies=	-2470.104918	$E_0 + G_{corr}$
Zero-point correction *(unscaled)* =	0.500155	

MO (number)	Energy, eV	Representation
LUMO+1 (152)	-1.096	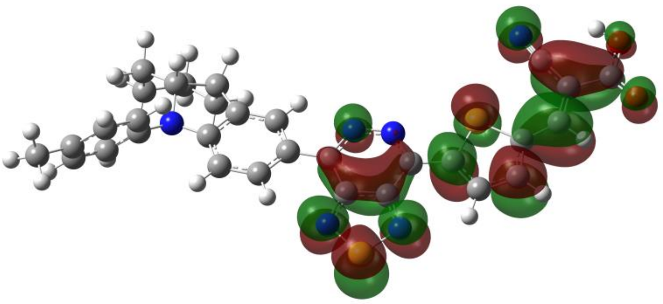
LUMO (151)	-2.024	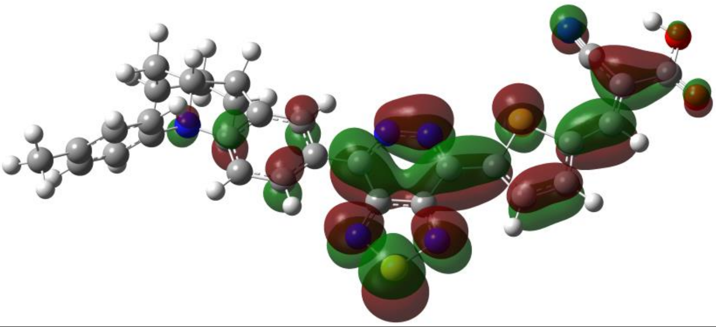
HOMO (150)	-7.383	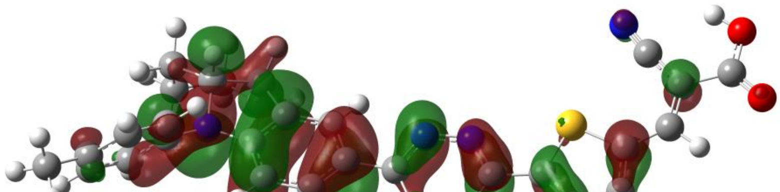

HOMO-1 (149)	-8.509	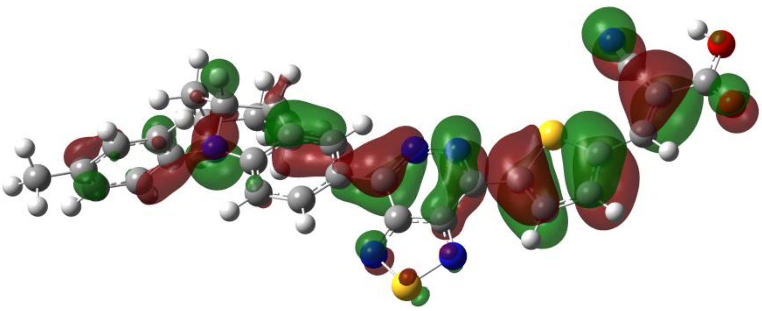
HOMO-7 (143)	-9.863	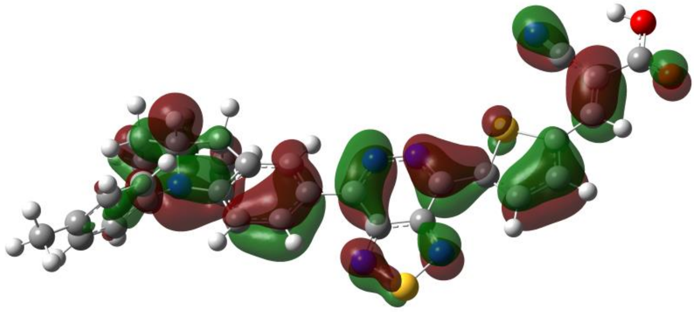

Calculated characteristics of the **first excited state** in gas phase. Vertical excitation, single point TD-DFT wB97XD/6-31+g(d',p') level of theory.

λ_{abs} vertical nm	f	Orbitals (contribution)	E_{gap} eV	μ S_0 D	μ S_1 vertical D	Δμ($S_{1\ vert}$-S_0) D
477	0.8792	HOMO-LUMO (83%)<br>HOMO-1-LUMO (7%)<br>HOMO-LUMO+1 (3%)<br>HOMO-7-LUMO+1 (2%)	5.36	11.06	11.06	0

RAW DATA for five lowest excited states

Excitation energies and oscillator strengths:

Excited State 1: Singlet-?Sym 2.6004 eV 476.78 nm f=0.8792 <S**2>=0.000

143 ->151 0.10343

149 ->151 -0.18984

150 ->151 0.64342

150 ->152 -0.12716

Total Energy, E(TD-HF/TD-DFT) = -2470.43688636

Excited State 2: Singlet-?Sym 2.9858 eV 415.25 nm f=0.0003 <S**2>=0.000

146 ->151 0.61414

146 ->152 -0.11000

146 ->154 0.10030

147 ->151 -0.19145

148 ->151 -0.19087

Excited State 3: Singlet-?Sym 3.5220 eV 352.03 nm f=0.4585 <S**2>=0.000

149 ->151 0.53024

149 ->152 0.22232

150 ->151 0.10568

150 ->152 -0.32553

Excited State 4: Singlet-?Sym 3.9304 eV 315.45 nm f=0.1321 <S**2>=0.000

149 ->151 0.34751

149 ->152 -0.36343

150 ->151 0.14184

150 ->152 0.38088

150 ->153 -0.13031

Excited State 5: Singlet-?Sym 4.1297 eV 300.23 nm f=0.0080 <S**2>=0.000

145 ->151 0.11907

146 ->151 0.23069

147 ->151 0.39424

147 ->152 -0.10639

147 ->153 -0.10035

148 ->151 0.37474

148 ->152 -0.12235

148 ->153 -0.11350

150 ->152 0.11017

150 ->160 0.10311
